# SiCmiR Atlas: Single‐Cell miRNA Landscape Reveals Hub‐miRNA and Network Signatures in Human Cancers

**DOI:** 10.1002/advs.202514446

**Published:** 2026-02-15

**Authors:** Xiao‐Xuan Cai, Jing‐Shan Liao, Jia‐Jun Ma, Yu‐Xuan Pang, Yi‐Gang Chen, Yang‐Chi‐Dung Lin, Yi‐Dan Chen, Xin Cao, Yi‐Cheng Zhang, Tao‐Sheng Xu, Tzong‐Yi Lee, Hsi‐Yuan Huang, Hsien‐Da Huang

**Affiliations:** ^1^ Warshel Institute for Computational Biology, School of Medicine The Chinese University of Hong Kong, Shenzhen Shenzhen Guangdong P. R. China; ^2^ School of Medicine The Chinese University of Hong Kong, Shenzhen Shenzhen Guangdong P. R. China; ^3^ Guangdong Provincial Key Laboratory of Digital Biology and Drug Development The Chinese University of Hong Kong, Shenzhen Shenzhen Guangdong P. R. China; ^4^ Department of Endocrinology Key Laboratory of Endocrinology of National Ministry of Health Peking Union Medical College Hospital Chinese Academy of Medical Sciences & Peking Union Medical College Beijing P. R. China; ^5^ Institute of Bioinformatics and Systems Biology National Yang Ming Chiao Tung University Hsinchu Taiwan

**Keywords:** atlas, biomarkers, cancers, hub‐miRNA, miRNA, single‐cell

## Abstract

MicroRNAs (miRNAs) are pivotal post‑transcriptional regulators whose single‑cell behavior has remained largely inaccessible due to technical barriers in single‐cell small‑RNA profiling. We present SiCmiR, a two‑layer neural network that predicts miRNA expression profiles from only 977 LINCS L1000 landmark genes, thereby reducing sensitivity to dropout in single‐cell RNA‐seq (scRNA‐seq) data. Proof‑of‑concept analyses illustrate how SiCmiR can uncover candidate hub‑miRNAs in bulk‐seq cell lines and hepatocellular carcinoma, scRNA‐seq pancreatic ductal carcinoma, and ACTH‑secreting pituitary adenoma and extracellular vesicle (EV)‑mediated crosstalk in glioblastoma. Trained on 6,462 TCGA paired miRNA–mRNA samples, SiCmiR attains state‑of‑the‑art accuracy on cancers and generalizes to unseen cancer types and drug perturbations. We next construct SiCmiR‑Atlas, containing 362 public datasets, 9.36 million cells, and 726 cell types, which is the first dedicated database of single‑cell mature miRNA expression, providing interactive visualization, biomarker identification, and cell‑type‑resolved miRNA–target networks. SiCmiR transforms bulk‑derived statistical power into a single‑cell view of miRNA biology and provides a community resource for biomarker discovery. SiCmiR Atlas is available at https://awi.cuhk.edu.cn/∼SiCmiR/.

## Graphical abstract

SiCmiR predicts mature miRNA activity at single‐cell resolution using only 977 landmark genes, enabling scalable reconstruction of miRNA landscapes across diverse tissues and disease contexts. The SiCmiR Atlas integrates 9.36 million cells with analytical tools for hub‐miRNA discovery, biomarker prioritization, and regulatory network interrogation, supporting both mechanistic insights and translational applications.

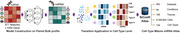

## 1 Introduction

MicroRNAs (miRNAs) are small non‐coding RNAs that regulate gene expression. Most function as repressors by binding to the 3'‐untranslated region (3’ UTR) of mRNAs, initiating mRNA degradation and blocking translation, though certain miRNAs have been reported to stabilize mRNA and to enhance its activity [1]. Dysregulation of miRNAs and their protein translation in the regulatory network underpins virtually every cancer hallmark, such as proliferation, stemness, invasion, and immune evasion. MiRNAs are therefore valuable biomarkers and therapeutic targets [2]. MiRNAs that are strongly associated with mRNAs play central roles in the regulatory network, and are referred to as hub‐miRNAs due to their significant impact [3]. As our understanding deepens, targeting these hub‐miRNAs offers a promising frontier for advancing both cancer diagnostics and therapy.

According to the records of miRBase, 2656 mature miRNAs have been identified in humans [4], although not all have been assigned functional significance. This knowledge gap may be attributed to factors such as inadequate sample sizes, sequencing batch effects, and limitations in capturing the true miRNA landscape [5]. Additionally, existing approaches for identifying disease‐related miRNAs, such as weighted gene co‐expression network analysis (WGCNA), which detects co‐expression patterns and hub nodes through topological overlap [6], or annotation‐based methods relying on miRNA‐target databases [7, 8, 9], suffer from incomplete functional annotations and limited coverage [10]. Machine learning and deep learning models have been introduced to predict disease‐related miRNAs based on known miRNA‐disease associations [11, 12]. However, challenges persist due to tumor heterogeneity [13], which complicates hub‐miRNA discovery.

While single‐cell RNA‐sequencing (scRNA‐seq) has significantly advanced mRNA‐level research, single‐cell miRNA sequencing remains underdeveloped with limited applications [14, 15, 16, 17]. However, existing single‐cell miRNA profiling techniques encounter several challenges, including dependence on polyadenylation, adaptor dimer formation, high data sparsity, difficulties in distinguishing miRNAs from other small non‐coding RNAs, and inconsistent protocol reproducibility [18]. These technical barriers have hindered the identification of hub‐miRNAs in cancers, emphasizing the need for improved methodologies. Recent efforts such as miRSCAPE [19] and miTEA‐HiRes [20] have markedly improved the recovery of miRNA activity at single‐cell resolution. miRSCAPE requires around 20 000 gene features and therefore suffers from zero inflation in scRNA‑seq. miTEA‐HiRes infers miRNA activity by testing target‐gene enrichment within spatial transcriptomic spots. It depends on canonical target lists, thus failing to capture continuous miRNA expression profiles.

**FIGURE 1:**
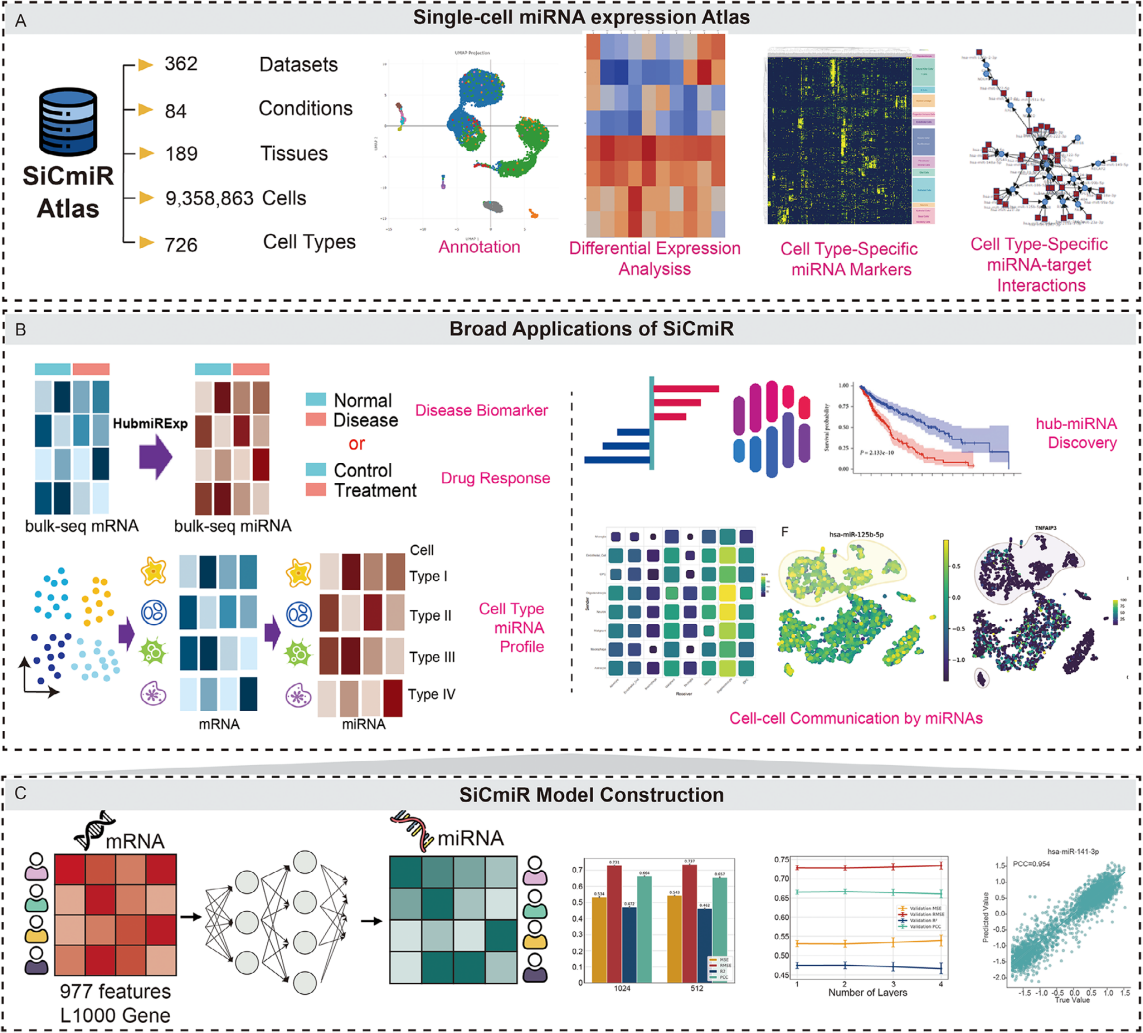
Schematic representation of the SiCmiR workflow, applications, and visualization. (A) Construction of the SiCmiR Atlas which archives inferred miRNA expression across diverse cell types, disease contexts, and four functional modules. (B) Multi‐task applications, including disease biomarker discovery, drug response prediction, cell‐type specific miRNA expression profile prediction, hub‐miRNA discovery, and miRNA‐mediated cell–cell communication demonstrated the broad utility of SiCmiR. (C) SiCmiR model architecture, leveraging a DNN to infer miRNA expression from landmark mRNA features.

SiCmiR addresses these limitations by relying on just 977 landmark genes and does not require prior knowledge of target genes. SiCmiR demonstrates its robustness in hepatocellular carcinoma, glioblastoma, and adrenocorticotropic hormone (ACTH)‑secreting pituitary adenoma (PitNET), and revealed 414 hub‐miRNAs in cancer, and also EV‐mediated intercellular communication at single‑cell resolution. To maximize the method's utility, we further compiled the predictions across multiple datasets as SiCmiR‑Atlas, an openly searchable database, the first public database dedicated to single‑cell mature miRNA expression, providing interactive visualization, biomarker mining, and cell‑type‑resolved miRNA–target networks. By coupling bulk‑derived statistical power with cell‐type level metadata, SiCmiR establishes a practical route to dissect miRNA regulation in heterogeneous tissues, thus accelerating biomarker and drug target discovery in oncology (Figure 1).

## 2 Results

### 2.1 Performance Analysis and Feature Selection of SiCmiR

#### 2.1.1 Performance Baseline Benchmarking and Feature Augmentation

**FIGURE 2:**
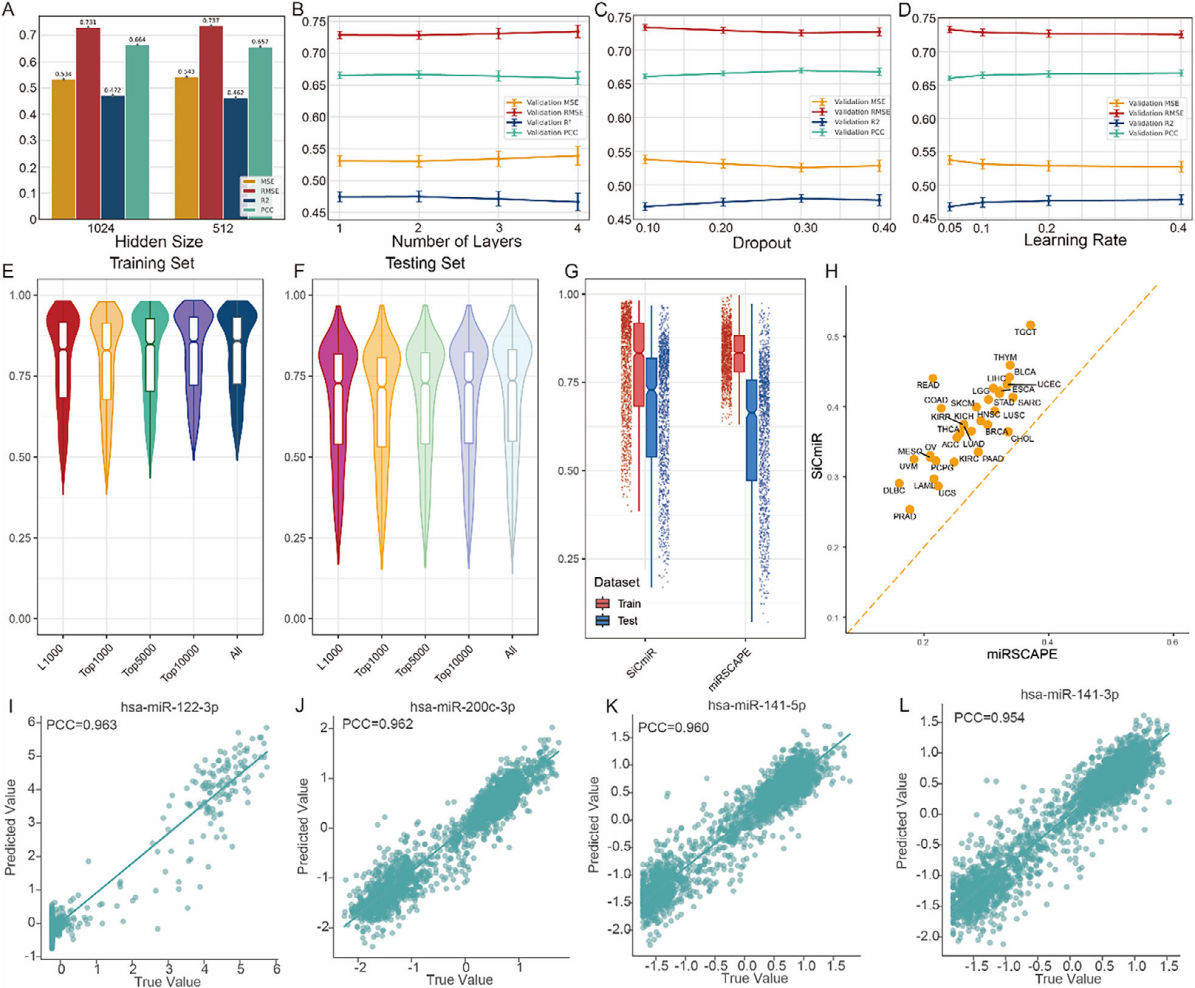
Performance evaluation on datasets. Mean square error (MSE), root mean square error (RMSE), Pearson correlation coefficient (PCC), and R^2^ were used for model performance evaluation. (A–D) compares the performance of models with the number of nodes in hidden layers set to 512 and 1024, the number of hidden layers ranging from 1 to 4, the dropout rate ranging from 0.1 to 0.4 in the SGD optimizer, and the learning rate ranging from 0.05 to 0.4. (E,F) Average PCC of each miRNA in the train set and test set using mRNA features of L1000 landmark genes, top 1000, 5000, 10 000 variable mRNAs, and all mRNAs. (G,H) Performance of SiCmiR excels miRSCAPE overall and across all cancer types. (I–L) High correlation between observed and predicted values in SiCmiR, highlighting miRNAs with the top performance.

The predictive performance of SiCmiR was evaluated by benchmarking multiple model architectures and validating the effectiveness of using 977 input features. Specifically, SiCmiR employs 977 landmark genes from the LINCS L1000 project (see Methods). These genes are recognized for their responsiveness to chemical and genetic perturbations, their high reproducibility across RNA‐seq datasets, and their capacity to infer approximately 81% of non‐measured transcript expression levels [21]. The use of these landmark genes also reduces data sparsity in single‐cell applications. Baseline performance was compared among neural networks, ResNet, and Transformer models. The neural network achieved superior performance (Table 1) and was consequently selected for subsequent optimization. Hyperparameters, including hidden layer size (1024 nodes), number of layers (2), dropout rate (0.3), and learning rate (0.4), were systematically tuned (Figure 2A–D). Using L1000 features, the mean Pearson correlation coefficient (PCC) across all miRNAs in the train and test sets reached 0.75 and 0.67, respectively (Figure 2E–F). Three‐fold cross‐validation confirmed the robustness of this performance, with average miRNA PCCs of 0.75 ± 0.00067 (train) and 0.67 ± 0.00073 (test), and sample‐level PCCs of 0.72 ± 0.09583 (train) and 0.63 ± 0.13707 (test) (Figure S1). To evaluate the sensitivity of model performance to input dimensionality, models were trained using different gene sets, including the 977 L1000 genes, and the top 1000, 5000, 10000, and all (*n* = 20062) variable protein‐coding genes. In comparison across feature sets, the average miRNA PCCs in the train set were 0.79 (L1000), 0.79 (top 1000), 0.81 (top 5000), 0.82 (top 10000), and 0.82 (all proteincoding genes). Performance of corresponding test set PCCs were 0.67, 0.66, 0.67, 0.67, and 0.68. Notably, when the number of features was limited to around 1000, the L1000 landmark genes consistently outperformed selected variable gene sets and offered substantial reductions in computational cost.

**TABLE 1:** The neural network model outperformed among basic models (Mean ± SE, 95% CI).

	MSE	RMSE	R2	PCC
**Neural Network**	**0.522 ± 0.0039 [0.5049, 0.5364]**	**0.722 ± 0.0027 [0.7107, 0.7326]**	**0.484 ± 0.0066 [0.4673, 0.4997]**	**0.673 ± 0.0023 [0.6635, 0.6818]**
ResNet	0.648 ± 0.0056 [0.6241, 0.6723]	0.805 ± 0.0035 [0.7901, 0.8200]	0.359 ± 0.0047 [0.3385, 0.3788]	0.578 ± 0.0024 [0.5678, 0.5882]
Transformer	0.863 ± 0.0077 [0.8301, 0.8962]	0.929 ± 0.0041 [0.9113, 0.9468]	0.146 ± 0.0016 [0.1390, 0.1530]	0.359 ± 0.0020 [0.3509, 0.3680]

SiCmiR also achieved higher predictive accuracy than miRSCAPE [19]. To ensure a fair and broadly applicable comparison, we retrained both frameworks on 6462 TCGA pan‐cancer samples. Using the 977 landmark genes, SiCmiR reached a PCC of 0.67 on an independent test set, outperforming miRSCAPE's 0.61 (Figure 2G). Notably, inference with a pre‐trained SiCmiR required only 2.23 s, whereas miRSCAPE required over 2 h due to its on‐the‐fly model training. When we adopted miRSCAPE's full‐gene feature space but unified training across cancer types, its pooled‐cancer model performance increased from the originally reported Spearman correlation coefficient of 0.62 to a PCC of 0.66 (Table S1). Despite this improvement, SiCmiR achieved comparable or slightly superior accuracy using an order of magnitude fewer inputs, highlighting its efficiency and robustness. Although cancer‐specific SiCmiR variants were also trained on individual tumor types to account for potential context‐dependent miRNA–mRNA regulation (Figure S2), the pan‐cancer SiCmiR model consistently outperformed these cancer‐specific models as well as miRSCAPE across multiple tumor types (Figure 2H; Figure S3) and was therefore selected for downstream analyses. For single‐miRNA performance, the highest predictive PCC was 0.984 for hsa‐miR‐21‐5p, as a well‐recognized oncogenic miRNA [22, 23]. Examples of true vs. predicted expression levels for four top‐ranked miRNAs are shown in Figure 2I–L, separately.

#### 2.1.2 Stress Testing Model Robustness Through Feature Loss and Sparsity

To assess whether SiCmiR depends on a limited subset of landmark genes, we performed stress tests including random feature dropout and SHAP‐guided feature removal. Randomly removing 5%–90% of L1000 landmarks resulted in a smooth and gradual drop in performance without abrupt performance collapse (Figure S4A). Even when roughly 70%–80% of genes were masked, the model maintained non‐trivial predictive accuracy. This pattern suggests that the model relies on a broad set of genes rather than a few indispensable ones. Collapse was defined as performance indistinguishable from an average predictor (R^2^ ≤ 0, PCC = 0), while non‐trivial accuracy was defined as PCC > 0.2 and R^2^ > 0 [24]. SHAP‐ranked ablations revealed a clear asymmetry. Removing features from low to high SHAP values led to a steady deterioration until more than 90% of the lowest‐ranked landmarks were removed, after which performance collapsed rapidly, indicating the presence of a compact high‐importance core (Figure S4B). Conversely, removing features from high to low in order of their SHAP values resulted in an almost perfectly linear deterioration across all metrics (Figure S4C). This result demonstrates that lower‐ranked features are not irrelevant. Their cumulative removal leads to a steady reduction of predictive strength rather than a flat plateau. Collectively, these results show that SiCmiR relies on both a compact core and a broad distributed tail of features, reflecting a non‐sparse, distributed representation rather than dependence on a small and isolated subset.

To evaluate whether SiCmiR remains stable under sparsity, a common challenge in single‐cell profiles, we subjected the matrix to three complementary perturbations. First, we applied uniform masking of non‐zero entries. Second, we introduced quantile‐stratified dropout using a Poisson‐based scheme with varying γ as the dropout rate, and the mean expression of genes was reported to follow a Poisson distribution [25]. Smaller γ approaches uniform masking and larger γ concentrates dropout in the lowest‐abundance entries. Third, we simulated sequencing‐depth reduction using multinomial UMI down‐sampling to mimic the dropout caused by capture rate following a binomial distribution [26], followed by Poisson‐based masking. Performance consistently declined as sparsity increased across all perturbations. Under uniform masking, our model collapsed by around 95% density (Figure S5A). Stratified Poisson‐based masking exhibited an intermediate effect, with performance degrading more gradually. When γ was large, dropout was concentrated in low‐expression genes, and the model's feature robustness, as mentioned, resulted in an apparent “inverse” behavior in which performance rose at extreme sparsity. In contrast, UMI down‐sampling displayed substantially greater stability (Figure S5B). Multinomial down‐sampling alone produced a decrease in performance as sequencing depth decreased. Notably, adding Poisson‐like masking (γ = 0.5) on top of UMI down‐sampling, sparsity reached 80%–90% without model collapse. Pre‐ and post‐z‐score normalization sparsity was tracked. Z‐scoring normalization reduced sparsity to zero across all sampling conditions without restoring performance. Collectively, these results indicate that depth reduction and masking disrupt the rank structure of gene expression, yet the model retains a non‐trivial predictive signal even in a challenging range of sparsity. For practical applications to real scRNA‐seq datasets, cell‐type‐averaged profiles or bootstrap‐pooled aggregations are recommended, as both approaches substantially reduce sparsity and improve the reliability of prediction.

### 2.2 Cross‑Cancer Generalization, Bulk‐Seq and scRNA‐Seq Case Studies

**FIGURE 3:**
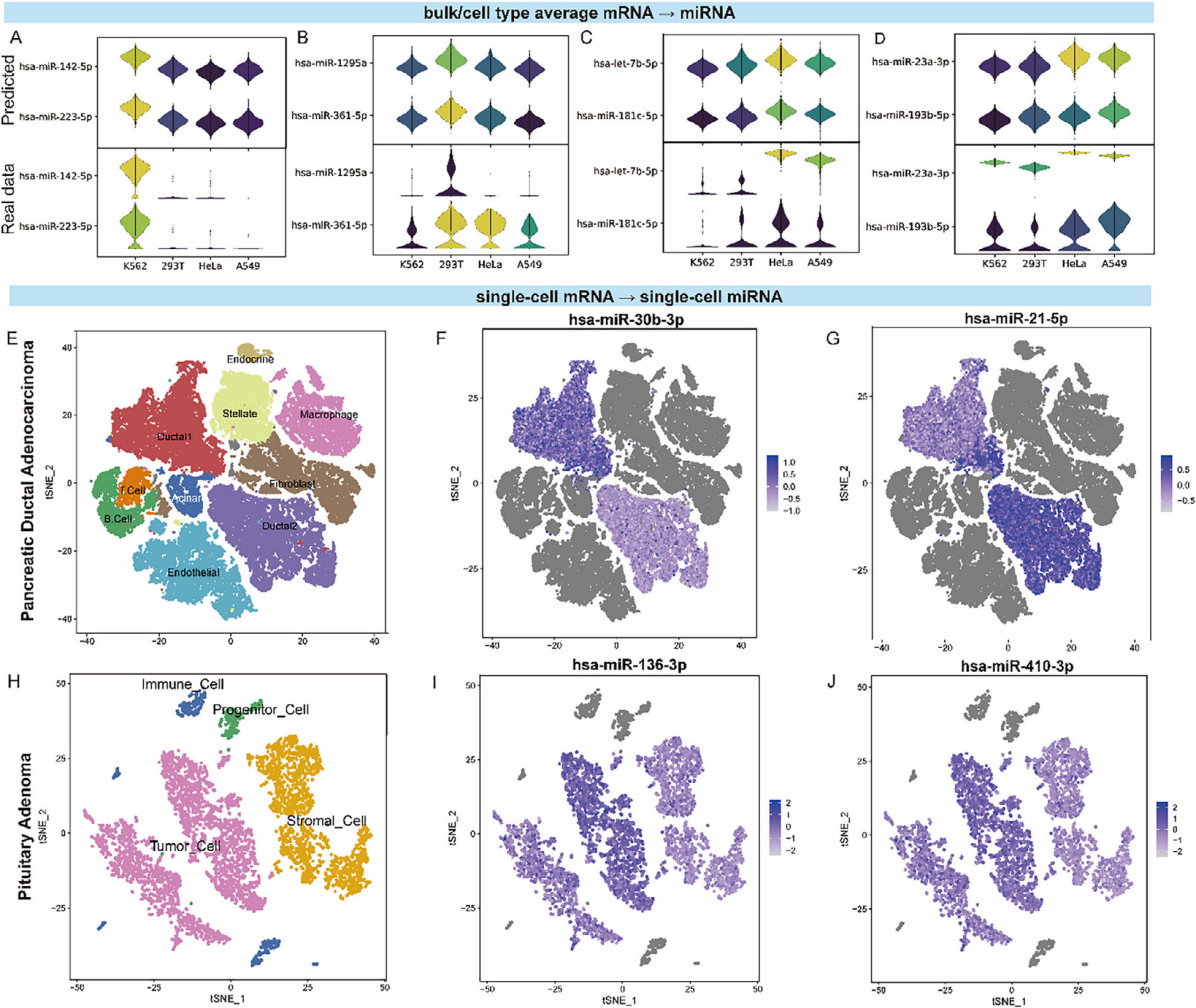
Expression prediction of miRNAs based on cell line RNA‐seq and scRNA‐seq data, consistent with literature on real datasets. (A–D) miRNA biomarkers of cell lines inferred from bulk RNA‐seq data (upper) show the same expression pattern as in bulk miRNA‐seq data (lower). (E) T‐SNE shows the annotation of cells in the PDAC scRNA‐seq dataset. (F–G) Feature plot of inferred expression profile of hsa‐miR‐30b‐3p and hsa‐miR‐21‐5p, showing significant difference in DC1, MUC5^+^ DC1, and malignant DC2. (H) T‐SNE shows cell annotation of cells in the PitNET scRNA‐seq dataset. (I,J) The feature plot of the inferred expression profile of hsa‐miR‐136‐3p and hsa‐miR‐410‐3p showed a significant difference between tumor cells and stromal cells, as in real data.

To assess the applicability of the proposed method to single‐cell miRNA expression prediction, immortal cell line data were first analyzed under the assumption of homogeneous expression across cells, such that bulk‐seq profiles represent average single‐cell expression. SiCmiR successfully predicted biomarker expression for K562, 293T, HeLa, and A549 cells (Figure 3A–D). Subsequently, SiCmiR was applied to scRNA‐seq data from pancreatic ductal adenocarcinoma (PDAC) samples collected by Peng et al. [27], comprising 57 530 cells: 41 986 from 24 PDAC samples and 15 544 from 11 normal tissues. Cell types were annotated using marker genes from the original study (Figure 3E; Figure S6A). Following the benchmarking strategy of Olgun et al. [19], a list of 101 dysregulated miRNAs from Mazza et al. [28] was used, of which 90 were present in the dataset. As type 1 ductal cells (DC1) are reported to be less malignant than type 2 ductal cells (DC2) [27], miRNA expression of DC2 within tumors was compared to that in DC1 and their precursor acinar cells. SiCmiR correctly predicted 66 miRNAs (sensitivity: 0.73) using pooled data and 28 miRNAs (0.31) using single‐cell data directly in DC2 cells compared to DC1 or acinar cells. Among the 39 dysregulated miRNAs assessable by miRSCAPE, SiCmiR provided predictions for 37 of them and correctly recovered 29 (0.78). For comparison, miRSCAPE, constrained to the same 977 landmark features as throughout subsequent analyses, correctly predicted 29 out of the full set of 39 (Table S2). Overall, SiCmiR achieved an accuracy of 0.65 (66/101), whereas miRSCAPE achieved an accuracy of 0.29 (29/101). Among them, hsa‐miR‐30b‐3p was highly expressed in DC1 compared to DC2 (Figure 3F), consistent with its higher expression in normal tissue compared to tumor tissues in bulk‐seq [28]. hsa‐miR‐21‐5p was overexpressed in DC2 and MUC5^+^ DC1 compared to MUC5^−^ DC1 (Figure 3G), in agreement with the reported cell‐type malignancy trajectory and negative association with clinical outcome [27, 29, 30]. Overall, the evaluation of the model on PDAC data demonstrates that our model enables the detection of miRNAs as biomarkers for distinguishing cell types and cell state differences in single‐cell sequencing data, achieving state‐of‐the‐art performance.

To further test the generalizability for cancer types not included in the TCGA data, we applied SiCmiR to ACTH‐secreting PitNET scRNA‐seq data from Zhang et al. [31], selecting two high‐quality samples (SRR13973073, SRR13973076). T‐SNE and marker‐based annotation from the original study identified 3314 tumor cells and 1948 stromal cells (Figure 3H; Figure S6B). Using stromal cells as baseline, SiCmiR predicted 55/75 reported dysregulated miRNAs with a sensitivity of 0.73 using pooled data and |log2FC| ≥ 0.25 [32, 33, 34, 35]. When raising the threshold to |log2FC| ≥ 1, sensitivity dropped to 0.53. The predicted expression of hsa‐miR‐136‐3p and hsa‐miR‐410‐3p (Figure 3I–J) was highly expressed in stromal cells rather than tumor cells, as expected [33]. Using non‐pooled single‐cell data, 46 miRNAs were predicted (0.61 sensitivity). Among these, 34 matched the expected trend (0.69) (Table S3). Among the top 414 miRNAs by prediction PCC ≥ 0.8, 49 were dysregulated, and 30/48 were predicted using cell‐type averages (0.625). These results demonstrate SiCmiR's applicability to cancer types not present in TCGA training data, which indicates that our model has learnt the miRNA expression pattern associated with mRNAs, and the application of our model is not restricted to cancer types of TCGA but also extends to a variety of cancer types.

SiCmiR also applies to bulk tissue and drug‐perturbed samples. For hepatocellular carcinoma (HCC) vs. normal tissue (*n* = 8), 19 of 24 DEmiRs from Varghese et al. [36] were recovered (sensitivity 0.79). Among 13 DEmiRs with PCC ≥ 0.8 in the test set of our model, 12 were correctly predicted (sensitivity 0.92), including hsa‐miR‐139‐3p/5p and hsa‐miR‐378d (Figure S7A). These results demonstrate SiCmiR's effectiveness in identifying DEmiRs in cancer tissue. We further applied SiCmiR to bulk RNA‐seq of A549 cells treated with *Cinnamomi Ramulus* (a traditional Chinese medicine) at three concentrations (*n* = 2) sequenced by our lab, with DEmiRs validated by qPCR [37]. Seven DEmiRs were identified, including five miRNAs (hsa‐miR‐25‐3p, hsa‐miR‐183‐5p, hsa‐miR‐218‐5p, hsa‐miR‐27a‐3p, hsa‐miR‐24‐3p) with PCC ≥ 0.8 in the test set. hsa‐miR‐218‐5p and hsa‐miR‐576‐5p were significantly downregulated, while hsa‐miR‐27a‐3p and hsa‐miR‐24‐3p showed decreasing trends with CR concentration (Figure S7B and Table S4). These results demonstrate SiCmiR's effectiveness in identifying drug‐perturbed DEmiRs.

To determine whether SiCmiR generalizes beyond cancer‐derived profiles, its performance was further evaluated in the GTEx cohort. This cohort provides paired mRNA–miRNA measurements generated from an independent sequencing center. Despite the substantial technical and biological shift between TCGA tumors and GTEx normal tissues, SiCmiR retained a PCC of 0.553 across miRNAs with PCC ≥ 0.8 and 0.465 on average among all miRNAs (Table S5). In contrast, miRSCAPE exhibited a marked loss of accuracy when the XGBoost model trained on TCGA was transferred to GTEx, with the average PCC falling below 0.25 across brain, breast, colon, liver, and lung tissue. These results suggest that SiCmiR captures a transferable component of the miRNA–mRNA regulatory structure that extends beyond cancer‐derived patterns.

### 2.3 SiCmiR Atlas Construction and Software Implementation

**FIGURE 4:**
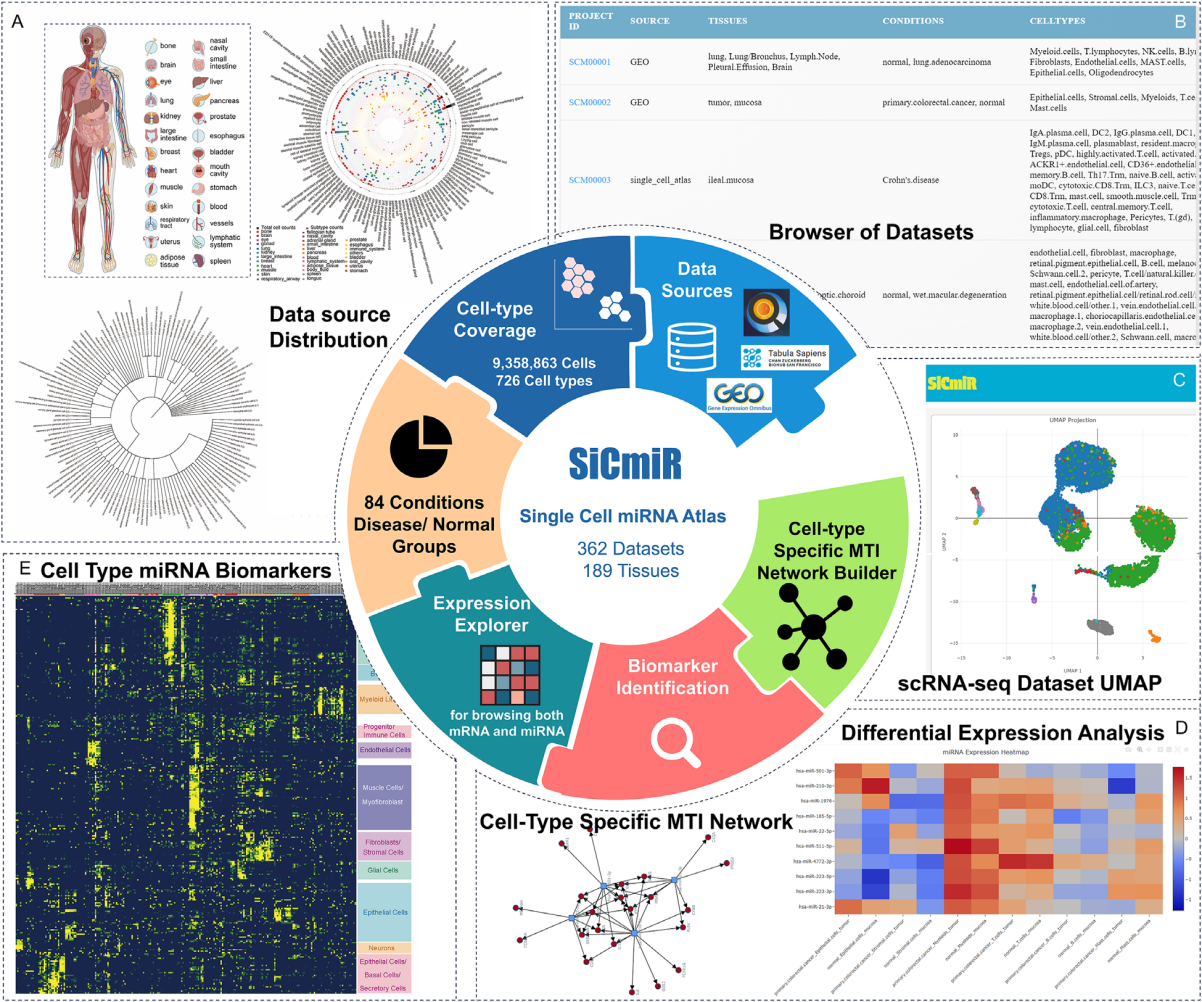
Architecture, data coverage, and core analysis modules of the SiCmiR atlas. (A) An overview of tissue type, cell type distribution, and the hierarchical clustering dendrogram of the cell population of data in the SiCmiR Atlas. (B) An example of a browser of datasets. (C) An example of UMAP demonstrating the cell type distribution of each dataset. (D) Function panel of differential expression analysis for miRNAs and mRNAs in each dataset to identify cell‐type specific biomarkers and to construct cell‐type specific MTI network interactively in the webpage. (E) Identified common cell type miRNA biomarkers by SiCmiR Atlas.

To demonstrate the utility of our method, we constructed the SiCmiR Atlas, which integrates 9.36 million single cells from 362 publicly available scRNA‐seq datasets spanning 189 anatomically distinct human tissues across 26 major organs, as defined by the Cell Ontology (Figures 1 and 4A) [38]. Cells annotated according to origin studies, yielding 726 unique cell identities from deeply embedded tissue‐specific sub‐types to broadly shared immune lineages. Clinical metadata were grouped into 84 physiological or disease conditions distributed over 12 broad disease categories. Based on this comprehensive resource, we implemented four fully integrated analysis modules: (i) data collection and annotation, storing matrices and cell‐type labels; (ii) biomarkers identification, providing interactive summaries of lineage representation across tissues and conditions; (iii) miRNA or mRNA differential analysis, supporting rapid visualization, expression comparison, and biomarker discovery for cell‐type‐enriched and disease‐associated miRNAs; (iv) in‐built MTI network builder infers miRNA–target interaction (MTI) graphs by integrating target‐site predictions by TargetScan [39], miRWalk [40], miRDB [41], and experimentally validated evidence from miRTarBase [42]. In particular, the differential analysis module supports contrastive analysis among cell types, allowing users to identify context‐specific dysregulation of miRNAs at single‐cell resolution across distinct cell types and disease contexts. Together, these results demonstrate that SiCmiR Atlas delivers a harmonized and annotated database of single‐cell miRNA biology. It supports interactive querying (Figure 4B), visualizes cell cluster distributions via UMAP (Figure 4C), and provides a coherent set of tools for interactive exploration, biomarker discovery, and construction of cell‐type‐resolved regulatory networks (Figure 4D,E). Notably, to our knowledge, SiCmiR Atlas represents the first publicly available resource dedicated specifically to single‐cell mature miRNA expression, providing a scalable, data‐driven foundation for both practical mechanistic studies and translational applications, including the development of diagnostic biomarkers and the prioritization of therapeutic targets, which are not supported by bulk‐based or ambiguously annotated datasets [43].

To further demonstrate the analytical capabilities of SiCmiR Atlas, cell type‐specific miRNA biomarkers were systematically identified across multiple tissues and conditions. Predicted expression profiles from 726 annotated cell types aggregated to prioritize miRNAs exhibited consistently high expression within specific lineages, including epithelial cells, endothelial cells, fibroblasts, oligodendrocytes, B cells, T cells/natural killer cells, myofibroblasts, neurons, and myeloid compartments, while remaining low expression in unrelated cell types. A representative heatmap (Figure 4E) presents a panel of cell‐type‐enriched miRNAs, revealing robust and recurrent expression patterns across diverse biological contexts. This analysis not only confirms established markers, such as hsa‐miR‐126‐5p in endothelial cells [44] and hsa‐miR‐141‐3p in epithelial cells [45] but also potentially uncovers novel candidates for cell identity and function. These conserved signatures offer a valuable reference for miRNA‐based cell‐type annotation, facilitate deconvolution of bulk miRNA data, and may serve as entry points for studying regulatory circuits in specific cellular compartments.

### 2.4 SiCmiR Discovered Hub‐miRNAs as Cancer Biomarkers

**FIGURE 5:**
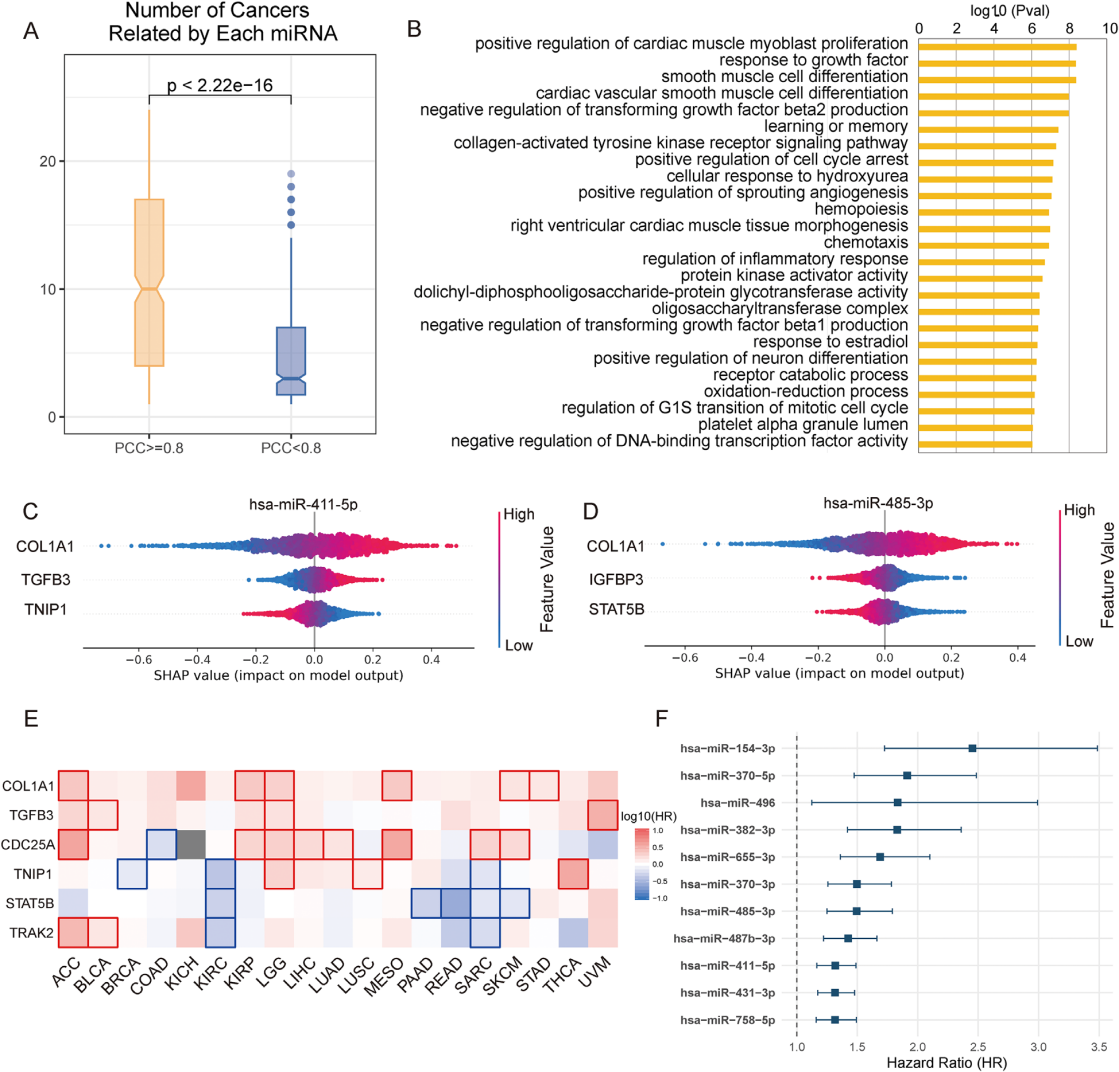
Identify hub‐miRNAs by SiCmiR. (A) Significant difference in the number of cancers associated with miRNAs with PCC ≥ 0.8 (*n* = 385) or PCC < 0.8 (*n* = 528), two‐tailed Wilcoxon rank‐sum test. (B) Top 25 GO enrichment analysis for miRNAs with PCC ≥ 0.8. (C,D) The contribution of landmark genes as features to the expression of miRNAs hsa‐miR‐411‐5p and hsa‐miR‐485‐3p. (E) Survival analysis of contributing features for their association with survival in cancers. (F) Survival analysis of miRNAs in module 10 for KIRP (*n* = 326).

The higher the correlation between miRNA and mRNA expression, the better the pattern of expression can be extracted, and the more active and tighter the regulation between them. An elbow‐like transition was observed at a PCC of 0.8 (Figure S8), above which miRNAs exhibited a pronounced increase in cross‐cancer involvement. Below this threshold, gains were limited. PCC = 0.80 was thus adopted as the threshold for hub‐miRNA identification. Subsequent enrichment and Shapley Additive exPlanations (SHAP) analysis was conducted to elucidate the role of the hub‐miRNAs selected. In the independent test set, 414 miRNAs satisfied this threshold, displaying reproducible expression profiles across 33 cancer types and implying tight regulation by their target mRNAs. Among these 414 miRNAs, 105 mature pairs (210 mature miRNAs) originate from the same pre‑miRNAs (for example, hsa‑miR‑141‑3p/5p), and many are members of the same families or primary transcripts, such as the miR‑200 and miR‑302 families. Compared to miRNAs of PCC < 0.8, these hub miRNAs formed denser cancer‑associated networks, with a mean degree of 11.12, approximately 2.4‑fold higher than that of other miRNAs (Figure 5A). Gene Ontology enrichment of their targets highlights pathways central to oncogenesis, progression, and metastasis (Figure 5B).

To interpret model predictions, SHAP was applied to quantify the contribution of each feature (Figure S9A) [46]. Network analysis of SHAP‑weighted edges revealed 12 functional modules (Figure S9B and Table S6). In module 10, which is driven by features *COL1A1, CDC25A*, and *GLI2*, miRNAs cluster on chromosome 14 (Figure S9C). Notably, 32 of 41 *COL1A1*‑contributing miRNAs, such as hsa‑miR‑127‑3p/5p, hsa‑miR‑134‑5p, hsa‑miR‑136‑3p/5p, are located on this chromosome. Target enrichment analysis links this module to metastasis‑related processes, including angiogenesis, extracellular matrix remodeling, and epithelial–mesenchymal transition (Figure S9D). For these 32 miRNAs, *COL1A1* exhibits a strong, positive, and significantly larger SHAP contribution compared to any secondary feature (Figure 5C–D; Figure S10). Survival analysis indicates that *COL1A1*, *TGFB3*, *CDC25A*, *TNIP1*, *STAT5B*, and *TRAK2*, the top contributors for these miRNAs, are correlated with prognosis in clear‑cell renal carcinoma (KIRC) and papillary renal carcinoma (KIRP) (Figure 5E), consistent with emerging reports [47, 48, 49, 50]. Consistent with these mechanistic insights, patients with elevated expression of the chromosome 14‐enriched miRNA set show markedly worse overall survival in the TCGA‐KIRP cohort, supported by hazard ratios consistently exceeding 1 under univariate Cox analysis (Figure 5F), which suggests that higher miRNA expression is associated with increased risk and worse prognosis. As renal‑cell carcinoma progression is dependent on angiogenesis, invasion, and migration, these findings align with the pathways enriched for *COL1A1*‑linked miRNAs and collectively demonstrate how model interpretation reveals hub‑miRNA/mRNA axes that drive cancer development.

### 2.5 SiCmiR Unlocks EV‑Mediated Communication Maps in Glioblastoma

**FIGURE 6:**
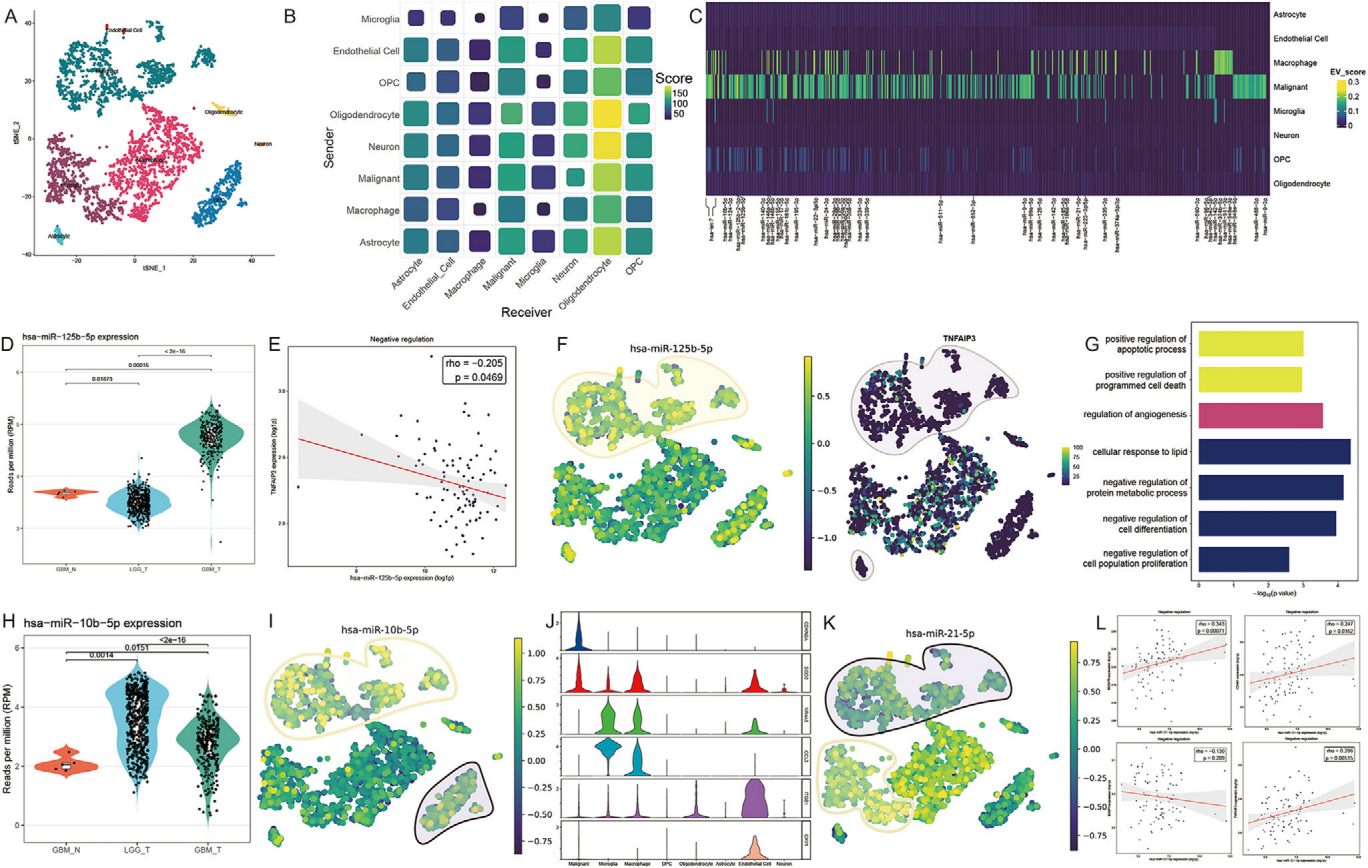
SiCmiR enables the unraveling of cell‐cell communication in GBM via EV‐mediated transfer. (A) T‐SNE embedding of 3497 cells clustered into eight lineages. (B) Sender‐receiver matrix summarizing cross‐talk strength. Size denotes the number of significant miRNA‐target edges, and color indicates the cumulative communication score. (C) Heatmap of EVmiR score of miRNAs denoting the abundance of across cell types. (D) Violin plot of hsa‐miR‐125b‐5p expression (log‐CPM) in TCGA‐GBM (*n* = 267), LGG (*n* = 530) vs. all non‐malignant tissue (*n* = 5), *p* value by two‐tailed Wilcoxon tests. (E) Spearman correlation between hsa‐miR‐125b‐5p levels and its targets *TNFAIP3* within the TCGA‐GBM cohort; shaded band, 95% CI. (F) Feature map: left, hsa‐miR‐125b‐5p; right, representative target gene *TNFAIP3*. Yellow indicates sender cells; purple indicates receiver cells. (G) GO enrichment for genes negatively correlated with hsa‐miR‐125b‐5p (‐log10 *p* value). (H) Expression landscape of hsa‐miR‐10b‐5p as (D). (I) Feature map for hsa‐miR‐10b‐5p with legend the same as (F). (J) Expression of five apoptosis‐related hallmarks as hsa‐miR‐10b‐5p target genes across cell types. (K) Feature map of hsa‐miR‐21‐5p, expression confined to malignant sub‐cluster. (L) Scatter plots showing Spearman correlations between hsa‐miR‐21‐5p and 4 canonical targets (*EGFR, MMP2, TGFBI, BTG2*) within the TCGA‐GBM cohort. Red lines indicate fitted regressions, shaded bands the 95% CI.

miRTalk [51] describes how EV‐small RNA cargo remodels the tumor niche by coupling a sender–miRNA “secreting score” with receiver cell RISC activity inferred from mRNA expression in a single‐cell profile. However, mature miRNA abundance usually does not align with miRNA gene expression (Figure S11), which limits this approach. To address this, we integrated SiCmiR‐inferred single‐cell miRNA profiles into the miRTalk framework and re‐evaluated cell‐to‐cell communication in glioblastoma (GBM). After quality control, 3,497 cells were retained (Figure 6A), identifying eight canonical lineages, including malignant cells, OPC‐like cells, and their brain‐resident stromal counterparts. Summarizing significant edges produced a sender‐receiver matrix (Figure 6B) that highlighted pronounced traffic from malignant cells and macrophages, whereas oligodendrocyte progenitor‐like cells (OPCs) and neurons primarily served as sinks. We identified 1 14 501 high‐confidence miRNA–target pairs (*p* < 0.05; Table S7). Incorporating SiCmiR‑inferred mature miRNA abundance into the miRTalk workflow significantly expanded both the breadth and biological coherence of the predicted EV‑mediated miRNA–target network (Table 2). This SiCmiR‑enhanced workflow yielded 1 14 501 high‑confidence miRNA–target interactions, larger than 20 times the original proxy analysis, and tripled the likelihood that an edge displayed the expected negative miRNA–mRNA Spearman correlation in the bulk RNA‐seq profiles in the TCGA‐GBM cohort. (36.9 % vs. 15.6 %; Fisher's OR  =  3.17, 95 % CI 2.94–3.42, *P* < 2.2 × 10^−^^1^ ^6^). The average interaction score increased nearly 50‑fold (0.04494 vs. 0.00095), reflecting a denser and more reliable interaction landscape. Consistent with the expectation that stronger interactions would translate into greater repression, the aggregate repression effect also slightly strengthened (Cliff's δ ‑0.21; one‑sided Wilcoxon *P* ≈ 0.011).

**TABLE 2:** SiCmiR Integration Dramatically Broadens and Refines EV‑Mediated miRNA–Target Networks.

Metric	miRTalk (gene‐proxy)	SiCmiR (mature‐miRNA)	Fold‐change / Gain	Statistical test
Total high‐confidence edges	5 390	114501	× 21.2	—
Negative‐correlated MTI (ρ < 0, *p* value < 0.05)	840	42273	× 50.3	Fisher exact test OR = 3.17 (95 % CI = 2.94‐3.42), *p* value < 2.2e‐16
Negative‐correlated MTI proportion	15.6 %	36.9 %	+21.3 pp	Same Fisher test as above
Repression effect size (Cliff's δ on receiver‐cell)	−0.2	−0.21	Slight increase	One‐sided Wilcoxon P = 0.0128, 0.0107
Average MTI score (by miRTalk)	0.00095	0.04494	× 47.23	Two‐sided Wilcoxon rank‐sum P < 2.2e‐16

A heatmap of sender scores ≥ 0.07 for individual miRNAs (Figure 6C) reveals marked cell‑type heterogeneity. At single‑miRNA resolution, SiCmiR identifies lineage‑restricted EV cargoes that modulate the tumor microenvironment. hsa‐miR‐125b‐5p was notably elevated in TCGA GBM samples compared to lower‐grade glioma (LGG) and non‐malignant cells (Figure 6D). Within TCGA‐GBM, miR‐125b‐5p levels correlated negatively with the expression of its validated targets (Figure 6E), indicating effective target repression in bulk tissue. These results were also reported by Shao et al. [51]. Feature overlays confirmed that miR‐125b‐5p is enriched in sender malignant clusters, while its target *TNFAIP3* is reciprocally expressed in malignant clusters and neighboring astrocytes (Figure 6F). These correlations supported an autocrine loop in which miR‐125b‐rich EVs reinforce lipid metabolism signaling and suppress apoptosis within the tumor core (Figure 6G) [52]. Likewise, hsa‑miR‑10b‑5p suppresses five pro‑apoptotic genes in recipient OPC‑like cells (Figure 6H–J), which are typically progenitors of malignant cells in GBM, consistent with previous reports that miR‐10b confers survival advantages and invasive phenotypes [53]. In contrast, macrophage‑enriched hsa‑miR‑21‑5p exports oncogenic signals to malignant clusters, correlating positively with *B3GNT5*, *ICAM1*, and *TNFAIP3* (Figure 6K,L). Overall, these results demonstrate that supplying mature‑miRNA expression predicted by SiCmiR not only increases network coverage but also substantially enhances the biological plausibility of miRTalk's intercellular miRNA–target interactions, providing a higher‑resolution view of EV‑mediated communication.

## 3 Discussion

This study introduces SiCmiR, a computational framework designed to infer miRNA activity from only 977 landmark genes and scales these predictions into SiCmiR Atlas, the first open repository of single‑cell mature‑miRNA landscapes. By shrinking the input space from more than 20 000 to 977 genes, SiCmiR mitigates zero‑inflation and attains state‑of‑the‑art accuracy across 33 TCGA tumor types and multiple scRNA‑seq datasets. SiCmiR Atlas warehouses predicted profiles, and cell‑type metadata in a user‐friendly webpage, enabling downstream applications interactively. Proof‑of‑concept case‐studies in HCC, GBM, and ACTH‑PitNET show that our framework recovers literature‑supported oncogenic miRNAs, reveals candidate hub regulators with prognostic value, and illuminates EV‑mediated crosstalk among malignant and stromal populations.

However, applying a bulk‐derived model to single‐cell data warrants careful interpretation. Biologically, bulk RNA‐seq captures population‐averaged transcriptional associations that may not fully reflect cell‐type‐specific or state‐dependent miRNA–mRNA quantitative relationships observable only at single‐cell resolution. Technically, single‐cell profiling introduces dropout and variable detection sensitivity that can weaken the coupling between true molecular abundance and observed expression values. These factors indicate that the accuracy of bulk‐trained models may be attenuated when biological heterogeneity or technical noise predominate. Sparsity remains another inherent challenge in single‐cell transcriptomics. Stress test results highlight the need for future noise‐aware architectures and sparsity‐robust feature priors. Notably, sampling from bulk FPKM shifts the retained‐gene composition toward lower‐expression ranks in higher UMI. This compositional shift coincides with the curve cross‐overs in Figure S5B, suggesting that the inversions arise from sampling‐induced changes in feature covariance structure rather than true model failure. This result highlights the value of incorporating raw count information during model training. Furthermore, we tracked sparsity before and after gene‐wise z‐scoring. As expected, z‐scoring maps zeros to finite values and eliminates numerical sparsity, but it cannot recover rank or covariance information lost during masking or down‐sampling, and model performance continued to follow the pre‐z‐score sparsity profile. Together, these results show that although SiCmiR remains stable under depth and sparsity reduction, cell‐type averages or bootstrap‐pooled profiles are recommended for application, as they substantially reduce sparsity and improve predictive reliability.

In our results, accurate predictions of mature miRNA levels and the observed correlation between mature miRNA expression levels within the same family are biologically expected [54, 55]. Members of a family often share substantial sequence similarity, which may result in overlapping mRNA target groups [56, 57], an aspect that aligns with the underlying rationale of our model design. In addition, members of the same miRNA cluster also originate from the same precursor primary transcript [58], reside in close chromosomal proximity, and share common transcription regulators [59]. These transcriptional and genomic relationships establish a biological backdrop that may contribute to the model's performance, even though it lacks explicit other translational information, such as allelic variation and modifications on DNA and histone, and also post‐transcriptional information such as pri‐miRNA structural constraints, Drosha/Dicer processing, RBP‐mediated stability control, and chemical modifications on nucleotides, all influencing splicing and degradation [60, 61].

On the other hand, our model is constrained by the fixed input feature space. Because SiCmiR relies on landmark genes, regulatory signals that arise from rare, lineage‐specific, or state‐dependent genes may remain under‐captured. These regulators are often lowly expressed in bulk but may exert a strong influence in single‐cell contexts [62]. While the current feature design supports robust generalization across datasets, future versions of SiCmiR could address this by integrating dynamic and context‐driven feature selection or adopting hybrid architectures that can flexibly embed variable‐length inputs while preserving computational efficiency. Refining the feature space may further enhance the performance and increase the resolution for mapping context‐specific miRNA regulation. Additionally, a more advanced model that integrates samples from both TCGA and GTEx could yield further improvements, as increasing sample size has been shown to enhance performance in both this and previous studies [19].

The case studies in our article show the utility of SiCmiR in identifying miRNAs with potential biological significance in cancers and cell types. By localizing dysregulated miRNAs to the cell types in which they are functionally active, SiCmiR enables researchers to better interpret miRNA‐driven processes during tumor development and progression. Among the miRNAs that were correctly detected by SiCmiR, hsa‐miR‐21, a well‐characterized regulator that suppresses apoptosis of tumor cells and promotes their proliferation [30], was consistently up‐regulated in all acinar cells, DC1, and DC2. hsa‐miRNA‐221, hsa‐miRNA‐222, and hsa‐miR‐146a, all of which have been implicated in promoting PDAC cell invasion [63], were upregulated in both DC1 and DC2 in cancer samples. Several dysregulated miRNAs identified by our framework have also been linked to tumor aggressiveness in recent literature. Among them, hsa‐miR‐147b‐3p was found to be significantly upregulated in DC2 cells in our analysis. Subsequent literature has reported its role in promoting invasion and proliferation [64, 65], aligning with our cell‐type level observations. Such convergence illustrates how SiCmiR can help pinpoint cell‐type‐specific miRNA dysregulation that may otherwise be obscured in bulk‐tissue analyses. This capability enables the identification of miRNAs with cell‐type‐restricted expression patterns, offering a more precise view of post‐transcriptional regulation across heterogeneous tissues. Meanwhile, we note that the CR‐A549 perturbation analysis illustrates the potential of SiCmiR for drug‐response profiling, but the small sample size warrants cautious interpretation. These results should be considered preliminary until validated in larger perturbation cohorts. Since SiCmiR is trained on standardized inputs, its outputs primarily reflect the relative ordering of miRNA abundance within a given cell type, rather than absolute expression scale. Expanding benchmarking to include absolute‐error metrics, calibration analyses, and perturbation‐response consistency will further clarify the quantitative fidelity of the predictions.

Beyond intracellular regulation, the application of SiCmiR improves miRTalk's basic strategy of predicting EV‐miRNA abundance, where the quantification implicitly assumes linear additivity and homogeneous vesicle uptake, it nonetheless inherits a key limitation of the original framework. Studies have demonstrated that EV‐miRNA loading is an active and selective process mediated by specific sequence motifs and RNA‐binding proteins such as hnRNPA2B1 [66], YBX1 [67], and SYNCRIP [68, 69], rather than a passive reflection of the intracellular miRNA pool. As a consequence, the current implementation does not explicitly model this selective packaging step. Although SiCmiR represents a substantial improvement over the previous use of host‐gene mRNA as a surrogate, the underlying assumption remains. Future efforts integrating EV‐specific sorting signals or context‐dependent EV‐miRNA profiles from bulk or, ideally, single‐cell datasets would be essential for capturing this additional layer of post‐transcriptional regulation.

Finally, we recognize the importance of long‐term maintenance and curation of the SiCmiR Atlas. The database will be updated regularly to incorporate new scRNA‐seq datasets, additional tissue contexts, emerging EV‐miRNA resources, and future model updates. Plans include refining the model structure, incorporating batch‐aware or domain‐specific strategies, and developing interactive tools that enable the community‐driven data calculation. Together, these efforts aim to ensure that SiCmiR Atlas remains an accurate, extensible, and evolving resource for studying miRNA regulation across diverse biological systems.

## 4 Conclusion

SiCmiR bridges a critical gap in single‐cell transcriptomics by enabling robust, fine‐grained inference of miRNA activity from a compact 977‐gene feature set. This approach mitigates dropout‐related noise that impairs transcriptome‐wide models and accelerates computation, thereby facilitating the routine integration of miRNA layers into single‐cell analyses. Scaling the pipeline across diverse publicly available datasets resulted in the creation of SiCmiR Atlas, a freely accessible repository that integrates predicted miRNA abundance with cell‐type annotations. Proof‐of‐concept studies in hepatocellular carcinoma, glioblastoma, and ACTH‐secreting PitNET demonstrate the resource's ability to uncover candidate hub‐miRNAs and to map extracellular‐vesicle‐mediated regulatory circuits at single‐cell resolution. Although several avenues remain for future refinement, the present work delivers both a methodological framework and a community resource that collectively establish a foundation for the systematic dissection of miRNA‐driven cell–cell interactions for advancing their translational application in precision medicine.

## 5 Methods

### 5.1 Data Collection for Model Training, Testing, and Case‐Study

Matched bulk log2(X+1) Fragments Per Kilobase of transcript per million mapped reads (FPKM) RNA‐Seq and reads per million mapped reads normalized (RPM) miRNA‐Seq gene expression data for cancers and normal samples were retrieved from TCGA using UCSC Xena at https://xenabrowser.net/ [70] (Figure S12). miRNAs were selected from the union of samples with at least one non‐zero expressing sample in the TCGA data. mRNA expression profiles of 977 mRNAs in 978 L1000 genes were extracted. XBP1 in L1000 landmark genes was excluded due to zero count in all samples. 1298 miRNAs out of 1952 miRNAs were selected to filter out miRNAs with zero counts in all samples. Known experimentally validated miRNA target information was gathered from the miRTarBase [42]. The training data set contains 6462 samples from 33 types of cancers. The rest of the samples from TCGA are used as independent validation sets. For independent validation datasets, there are totally 2768 samples. The ratio of the number of samples for each cancer type is around 3 to 1 as the ratio of the number of samples in the training dataset and test dataset is close to 3:1.

For case‐study, bulk RNA‐seq and small RNA‐seq data were collected. Bulk RNA‐seq data for hepatocellular carcinoma was collected from Varghese et al., GEO accession number: GSE176289 [36]. Bulk RNA‐seq data for the non‐small cell lung cancer (NSCLC) A5459 cell line is generated by our lab and published by Li et al. [37]. The scRNA‐seq expression profile of GBM obtained from GEO with accession GSE64465 [71]. The expression of each mRNA and miRNA across cells was normalized by z‐score across samples before applying the SiCmiR model. The scRNA‐seq expression profile for PDAC was collected from Peng et al. [27]. at PRJCA001063 from https://ngdc.cncb.ac.cn/. The PitNET scRNA‐seq data is retrieved from GEO with accession SRR13973073, SRR13973076. Cells were filtered by nFeature_RNA ≥ 200 and percentage of mitochondrial reads ≤ 10%. Gene counts were library‐size normalized (CPM × 1e4) and log‐transformed. For GTEx data, 15398 paired samples within Transcripts Per Million (TPM) RNASeQCv2.4.2 mRNA matrix and miRNA TPM matrix are retrieved from the GTEx portal at https://www.gtexportal.org/.

### 5.2 Machine Learning Model for miRNA Profile Prediction

We have adopted the neural network architecture that predicts miRNA profiling based on the given mRNA expression levels. Denote the training dataset $D=\{\left(x^{\left(1\right)},y^{\left(1\right)}\right),\text{…},\left(x^{\left(N\right)},y^{\left(N\right)}\right)\}$ with a total of *N* samples, where the $x^{\left(n\right)}\in R^{d}$ stands for the *d*‐dimensional gene expression vector and $y^{\left(n\right)}\in R^{m}$ represents the *m*‐dimensional vector of miRNA profiling values for the *n*‐th sample. The goal is to utilize D to learn a neural network‐based multi‐target repression model $F_{\theta }\left(\cdot \right)$ parameterized by θ that maps the input gene expression vector *x* to the output vector $\hat{y}$ of the miRNA values. A two‐layer fully connected network (input = 977, hidden = 1024, output = 1298) was implemented. Hyper‐parameters were tuned by grid search (Table S8). Early stopping after 20 epochs without validation loss improvement. We considered batch normalization [72], dropout [73], and rectified linear unit (ReLU) [74] for each hidden layer to avoid overfitting and improve the prediction performance. A detailed schematic diagram for the structures of the adopted neural network model, and ResNet and Transformer model for comparison is illustrated in Figure S13 and Supporting Information 8. 20% of the training set is separated randomly by seed = 42, 52, 62 as a validation set. To achieve the predictive performance of the regression tasks, the model utilizes a mean squared error (MSE) loss function *l*(·) as Equation (1).

(1) $$l\;\left(D,\;\theta \right)=\frac{1}{N}\;\sum_{n=1}^{N}∥y^{\left(n\right)}-{\hat{y}}^{\left(n\right)}∥$$

where the ${\hat{y}}^{\left(n\right)}$ denotes the output prediction for the *n*‐th training sample. The supervised loss encourages the model parameter θ to update and finally be capable to predict miRNA values from gene expression inputs. We optimized the model training by tuning the stochastic gradient descent optimizer with a 0.4 learning rate. The dropout rates are set as 0.3 for the hidden layers. In addition, we performed stratified 3‐fold cross‐validation in the training data set to further test the stability of the model. Feature selection (977‐gene landmark) was fixed a priori, and thus, cross‐validation was not nested.

### 5.3 Performance Evaluation

To characterize the predictive performance of our proposed regression model, we adopted the Pearson correlation coefficient (PCC) to measure the consistency between model prediction and the ground‐truth miRNA prediction value. The PCC for a specific miRNA regression is defined as Equation (2).

(2) $$\mathrm{PCC}=\frac{\sum_{n=1}^{N}{(y}_{k}^{\left(n\right)}-\bar{y_{k}}{)(\hat{y}}_{k}^{\left(n\right)}-\bar{{\hat{y}}_{k}})}{\sqrt{\sum_{n=1}^{N}{{(y}_{k}^{\left(n\right)}-\bar{y_{k}})}^{2}\sum_{n=1}^{N}{{(\hat{y}}_{k}^{\left(n\right)}-\bar{{\hat{y}}_{k}})}^{2}}}\;$$

where the $y_{k}^{\left(n\right)}$ and ${\hat{y}}_{k}^{\left(n\right)}$ stand for *k*‐th miRNA ground‐truth value and the prediction result of the *n*‐th sample from the dataset. The $\bar{y_{k}}=\frac{1}{N}\;\sum_{n=1}^{N}y_{k}^{\left(n\right)}$ and $\bar{{\hat{y}}_{k}}=\frac{1}{N}\;\sum_{n=1}^{N}{\hat{y}}_{k}^{\left(n\right)}$ are the average of true miRNA profiles and prediction value, respectively. Mean square error (MSE, the same as the loss function) Equation (3) and root mean square error (RMSE) Equation (4) quantify the average squared and root‐squared deviations, with RMSE sharing the same scale as the original data.

(3) $$\mathrm{MS}E_{k}=\frac{1}{N}\;\sum_{n=1}^{N}{\left({\hat{y}}_{k}^{\left(n\right)}-y_{k}^{\left(n\right)}\right)}^{2}\;$$

(4) $$\mathrm{RMS}E_{k}=\sqrt{\mathrm{MS}E_{k}}\;$$

The coefficient of determination R‐square (R^2^) Equation (5) revaluates how much of the variance in the ground‐truth expression levels is explained by the model where *R* ^2 ^ =  1 indicating perfect fit, *R* ^2^ =  0 means the model performs no better than simply predicting the mean, and *R* ^2^ < 0 implies worse performance than the mean predictor.

(5) $$R_{k}^{2}=1-\frac{\sum_{n=1}^{N}{{(\hat{y}}_{k}^{\left(n\right)}{-y}_{k}^{\left(n\right)})}^{2}}{\sum_{n=1}^{N}{{(y}_{k}^{\left(n\right)}{-\bar{y}}_{k}^{\left(n\right)})}^{2}},\;\;{\bar{y}}_{k}=\frac{1}{N}\;\sum_{n=1}^{N}y_{k}^{\left(n\right)}$$

Model training was performed on an NVIDIA A100 PCIe GPU (40 GB VRAM) in a workstation equipped with an Intel Xeon Gold 6326 CPU and 251 GB memory. The software environment required PyTorch 1.13.0+cu117 (CUDA 11.7), NumPy 1.26.4, pandas 2.0.3, and scikit‐learn 1.7.1. Under this configuration, one training epoch required approximately 0.33 s, yielding a total wall‐clock time of approximately 70 s for 200 epochs, with minor variation depending on GPU load. These hardware and software specifications support the reproducibility of the training setup.

### 5.4 Feature Ablation Stress‐Test Framework for Evaluating Model Robustness

All perturbations were applied directly to the input matrix used for model prediction, and SiCmiR was run without any modification to architecture or training parameters to ensure that changes in performance reflected only perturbation effects. Random feature dropout: In each iteration, a fixed percentage of landmark genes ([0, 1, 5, 10, 20, 30, 40, 50, 60, 70, 80, 90]) was masked by setting their expression values to zero for all samples. The model was then evaluated on 6 different random seeds [101, 204, 387, 1567, 640, 912] across dropout levels, allowing us to quantify stability as feature availability decreases. SHAP‐guided feature removal: feature landmarks were ranked by their average SHAP contributions to hub‐miRNAs. Two cumulative removal series were generated. In each series, the top *k* (or bottom *k*) genes were masked jointly at step *k*, rather than removed individually, generating a progressive increase in the fraction of eliminated features. Model performance was evaluated at every cumulative removal level.

### 5.5 Sparsity Stress‐Test Framework for Evaluating Model Robustness

Bulk RNA‐seq expression values, reported as log_2_(expression+1), were first converted back to an expression matrix of 977 landmark genes, and were perturbed to assess model robustness under increasing sparsity. Matrix was then z‐scored across samples and input to the model. To mimic a simple increase in dropout, we uniformly masked a proportion of non‐zero entries across the entire matrix. For each sparsity percentage ranging [0, 0.1, 1, 5, 10, 20, 30, 40, 50, 60, 70, 80, 90], a random subset of existing non‐zero counts were set to zero. The evaluation was repeated by 6 different random seeds [62, 567, 309, 39, 675,^42^] across sparsity levels. Poisson‐based sampling mimic the distribution in which the probability of dropout increases as the expression of gene decreases [25]. For each non‐zero entry indexed by Equation (6).

(6) $$\left(i,g\right)\in \Omega =\{\left(i,g\right):\mathrm{FPK}M_{\mathrm{ig}}>0\}$$

where *i* denotes a sample and *g* denotes a gene, we defined dropout probability *p_ig_* as Equation (7).

(7) $$\begin{array}{ccc} p_{ig} & = & exp\left(-\gamma \lambda_{ig}\right)\;where\;\lambda_{ig}\\ & = & \max\left(\frac{FPKM_{ig}}{percentile_{95}\left(FPKM_{ig}\right)},\;{10^{-}}^{12}\right)\; \end{array}$$

Each entry was then independently masked by a Bernoulli distribution as Equation (8).

(8)

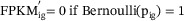




To approximate reduced sequencing depth, we applied multinomial sampling to every sample, since the sequencing depth is determined by the capture rate following binomial distribution [26]. For each sample *i*, the probability vector *p_i_* was normalized to a probability distribution Equation (9).

(9) $$p_{i}=\frac{\mathrm{FPK}M_{\mathrm{ig}}}{\sum_{g}\mathrm{FPK}M_{\mathrm{ig}}}\;$$

In order to clarify the effects of sparsity rather than feature dropout, γ  =  0.5 was selected in the next step. A pseudo‐UMI counts reduced were generated by multinomial sampling by Equation (10).

(10)

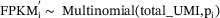




Reduced sequencing depth was then simulated by Poisson‐based sampling as above with a dropout probability 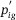 and sparsity after down‐sampling *sp_down_* to satisfied the target sparsity *sp_target_* as Equation (11).


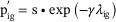

(11) $$\begin{array}{ccc} \mathrm{where}\;s & = & \frac{{\mathrm{sp}}_{\mathrm{target}}^{'}}{\bar{p_{\mathrm{ig}}}}\;,\;\;\bar{p_{\mathrm{ig}}}=\frac{1}{\left|\Omega \right|}\;\sum_{\left(i,g\right)\in \Omega }p_{\mathrm{ig}},\\ \mathrm{and}\;{\mathrm{sp}}_{\mathrm{target}}^{'} & = & \frac{{\mathrm{sp}}_{\mathrm{target}}\;-\;{\mathrm{sp}}_{\mathrm{down}}}{1\;-\;{\mathrm{sp}}_{\mathrm{down}}}\; \end{array}$$

### 5.6 SHAP Analysis Attributes the Contribution of Each mRNA to Output

To attribute the contribution of each input feature to the model output, gradient explainer for SHAP analysis was adopted [46]. The average contribution of each feature to each output miRNA in each paired sample was calculated.

### 5.7 Annotation of miRNA Functions and Pathway Enrichment Analysis

miEAA2 (https://www.ccb.uni‐saarland.de/mieaa2) was used for the annotation of miRNAs of their roles in different types of cancers [75]. Enrichment analysis of miRNAs was also conducted. The over‐represented mode was chosen for the annotation of miRNAs in cancers. The network graphs between miRNAs and cancers and the analysis of the network, e.g., degree of nodes, were plot and calculated by Gephi [76]. MetaCore (@Clarivate Analytics, https://portal.genego.com/) and gene ontology [77] by R package clusterProfiler [78], and Kyoto Encyclopedia of Genes and Genome (KEGG) Pathway database [79] was used for gene enrichment analysis. Default parameters were used for analysis.

### 5.8 Survival Analysis for Discovered Hub‐miRNA

Survival analyses for miRNAs in interests in cancers were performed by the univariate Cox proportional Hazard model which quantifies the change in hazard associated with a one‐unit increase in miRNA expression. Survival analysis for mRNA in cancers are computed by GEPIA2 [80]. Difference of survival rate between m low and high expression with *p* value <0.05 was regarded as significant.

### 5.9 Data Processing and Differential Expression Analysis

DESeq2 [81] with *p* value calculated by Wald test were used for differential expression analysis for bulk‐seq/predicted‐bulk miRNA expression profile. For predicted single‐cell miRNA expression profiles, Seurat V4 [82] was used to conduct differential expression analysis with *p*‐value calculated by the Wilcoxon Rank Sum test (Wilcox, *p*‐value <0.05).

### 5.10 scRNA‐seq Data Sampling and Pooling for Case‐Study

In the case‐study part, scRNA‐seq data were sampled by cells in each reported cell types and pooled as pseudo‐bulk data for better prediction accuracy. The scRNA‐seq data was pooled in order to avoid the sequencing bias and sparsity of scRNA‐seq or conducted cell type average pooling. For pooling average, cells in each cell type are randomly sampled not replacing 80% of cells for average pooling in one pooled sample, which is the same as the bootstrapping method in Olgun et al. [19].

### 5.11 Cell–Cell Communication Imputation

miRTalk [51] algorithm is used for imputations with miRNA host genes expression replaced by predicted miRNA expression profiles by SiCmiR. Parameters remaining default.

### 5.12 Database Implementation and GitHub Usage

All datasets included in SiCmiR Atlas were curated from Single Cell Atlas, Tabula Sapiens, and GEO under strict and fully reproducible criteria. Only Homo sapiens, UMI‐based datasets with publicly available raw count matrices with more than 500 cells, and metadata specifying tissue of origin and disease state were retained, whereas non‐human, mixed‐species, and metadata‐insufficient datasets were excluded. To ensure consistency, all datasets underwent a unified stringent QC workflow in which cells were retained only if nFeature_RNA > 200 and < 2500, percent.mt < 5%. To avoid cross‐study batch artifacts, raw count matrices were never merged. Instead, preprocessing, SiCmiR inference, and downstream visualizations were performed independently per dataset, ensuring that observed patterns reflect biology rather than technical drift. datasets with more than 400 cells per cell type underwent bootstrap sampling to obtain stable pooled estimates; otherwise, cell‐type averages were used. The sparsity of the input data was thus reduced (Figure S14). Datasets containing only two cell types that did not meet these criteria were excluded because averaging followed by z‐scoring would yield degenerate constant values. Cell annotation followed a two‐step procedure based on original metadata and subsequent harmonization for atlas‐level statistics and visualization. The atlas webpage was implemented using the Apache Wicket framework on a local CentOS high performance computational server, and the SiCmiR model is openly available on GitHub at https://github.com/Cristinex/SiCmiR/. For the visualization of predicted miRNA level in different subpopulation, the R package Seurat V4 [82] was used. SiCmiR accepts gene‐by‐cell expression matrices in CSV format and cell‐by‐gene matrices in h5ad format following the default AnnData orientation. Inputs must be annotated with gene symbols. Missing L1000 landmarks are filled as 0. The package outputs per‐cell, cell‐type average, or bootstrap‐pooled mature‐miRNA predictions, as specified by the user. SiCmiR runs efficiently on CPU. The main constraints arise from memory load when extracting landmark genes from very large matrices and from the bootstrapping step used for cell sampling. When users provide a pre‐subset matrix and bootstrapping is not required (although strongly recommended for datasets with very large cell numbers), inference is extremely fast and scales well on standard workstations. An interacting web interface is under development to further support community use.

### 5.13 Statistical Analysis

All statistical analyses relevant to each experiment or comparison are described in the corresponding Method subsections and figure legends, including data preprocessing, sample sizes, and the specific tests applied. Unless otherwise noted, two‐sided tests were used with a significance threshold of *p*‐value = 0.05. Computational analyses were performed using Python and R with standard libraries, complemented by web‐based tools mentioned where appropriate.

## Funding

This research was funded by Shenzhen Science and Technology Innovation Program [JCYJ20220530143615035, JCYJ20250604141235046, JCYJ20250604141041017]; Guangdong S&T Programme [2024A0505050001, 2024A0505050002]; Warshel Institute for Computational Biology funding from Shenzhen City and Longgang District [LGKCSDPT2025001]; Guangdong Young Scholar Development Fund of Shenzhen Ganghong Group Co., Ltd. [2021E0005, 2022E0035, 2023E0012].

## Conflicts of Interest

The authors declare no conflicts of interest.

## Supporting information




**Supporting File 1**: advs73630‐sup‐0001‐SuppMat.docx.

**Supporting File 2**: advs73630‐sup‐0002‐Data.zip.

## Data Availability

The authors declare that data supporting the findings of this study are available within the article and supplementary information. Package for usage of SiCmiR can be retrieved at https://github.com/Cristinex/SiCmiR. Online application is deposited at https://awi.cuhk.edu.cn/~SiCmiR/. The data that support the findings of this study are openly available in Zenodo at https://doi.org/10.5281/zenodo.16025109.
