# Supplementary material for: SiCmiR Atlas: Single‐Cell miRNA Landscape Reveals Hub‐miRNA and Network Signatures in Human Cancers
Source: Adv Sci (Weinh). 2026 Feb 15;13(16):e14446. doi: 10.1002/advs.202514446 (PMC13042402; doi:10.1002/advs.202514446)
Supplement: Supplementary file 1 — Supporting File 1: advs73630‐sup‐0001‐SuppMat.docx. [file ADVS-13-e14446-s002.docx]

Supporting Information

SiCmiR Atlas: Single-Cell miRNA Landscape Reveals Hub-miRNA and Network Signatures in Human Cancers

Xiao-Xuan Cai^1,2^, Jing-Shan Liao^2^, Jia-Jun Ma^2^, Yu-Xuan Pang^1^, Yi-Gang Chen^1,2^, Yang-Chi-Dung Lin^1,2,3^, Yi-Dan Chen^1,2^, Xin Cao^2^, Yi-Cheng Zhang^2^, Tao-Sheng Xu^1^, Tzong-Yi Lee^5^, Hsi-Yuan Huang^1,2,3,*^, and Hsien-Da Huang^1,2,3,4,*^

**
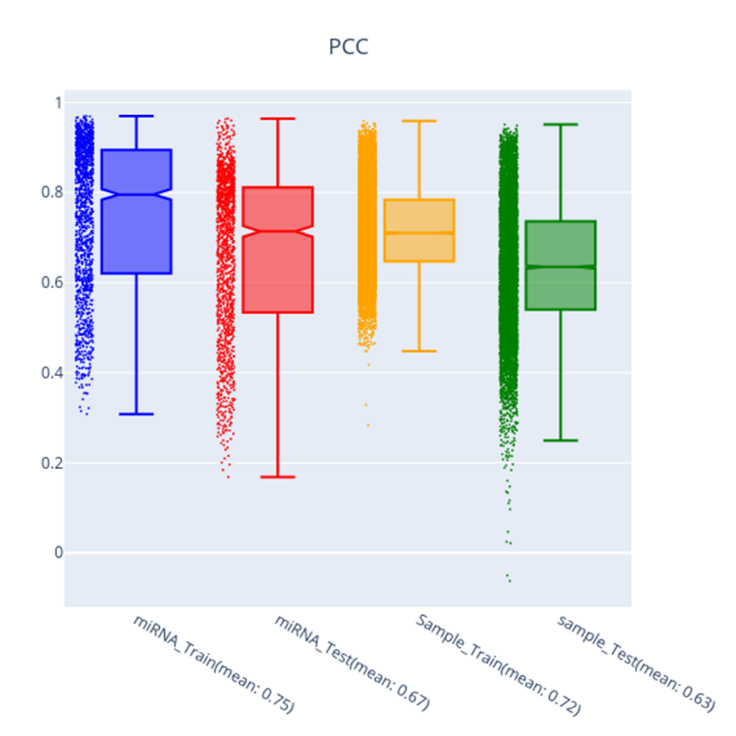
**

**Figure S1. Box plot for model performance by 3-fold cross-validation.** (Blue and red) Training and test dataset average PCC of miRNA among all samples. (Yellow and Green) Boxplot of average PCC of samples among all miRNAs in the training and test dataset, respectively.


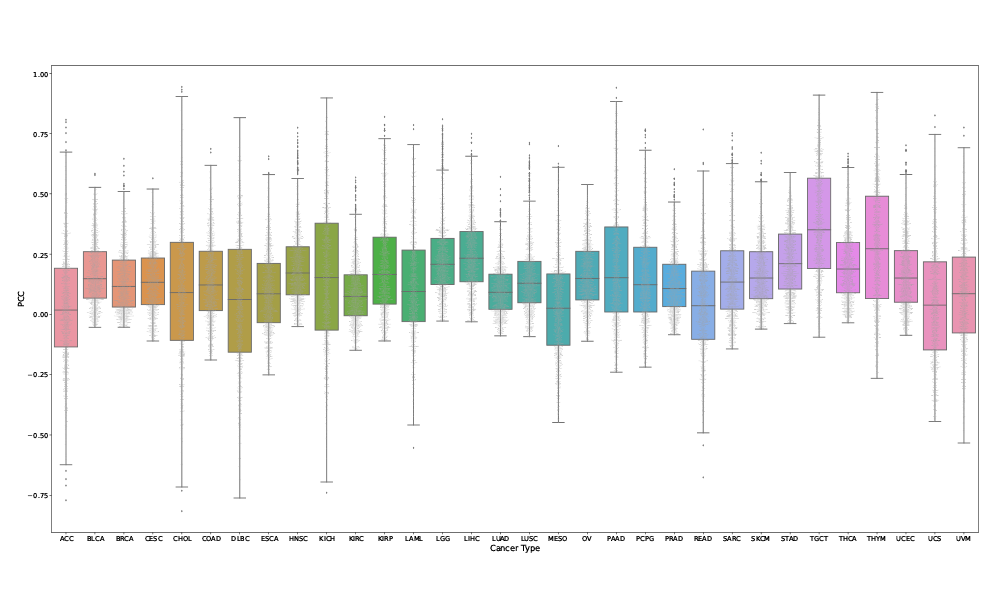


**Figure S2. Box plot for performance of cancer-type specific models.**


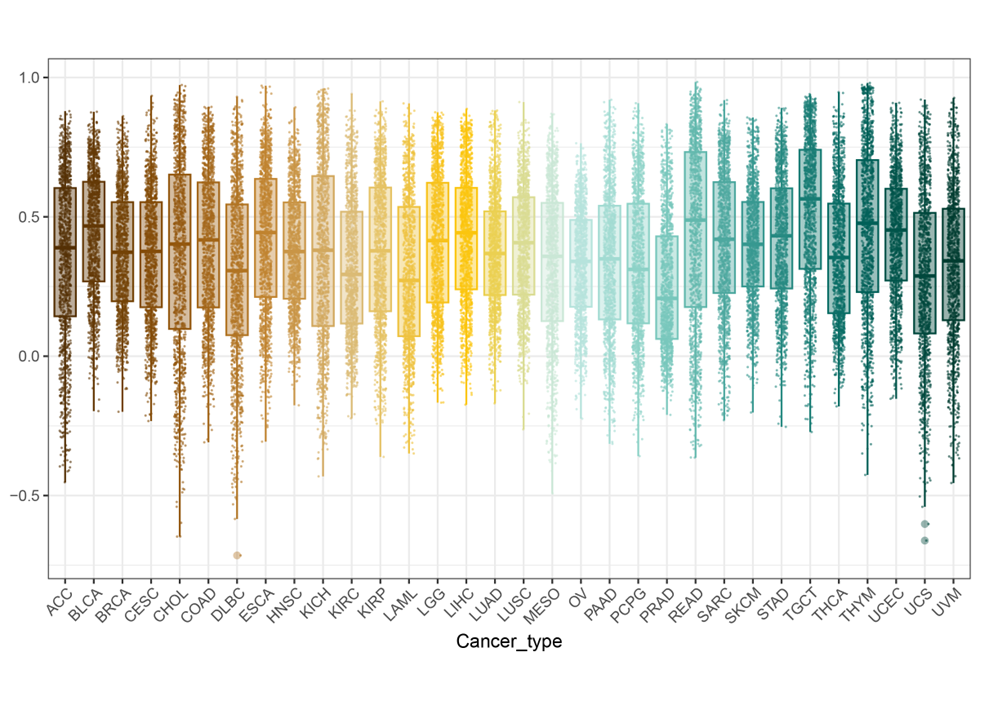


**Figure S3. Average model performance (PCC) of each miRNA in different cancer types predicted by model trained with all types of cancers.**

**
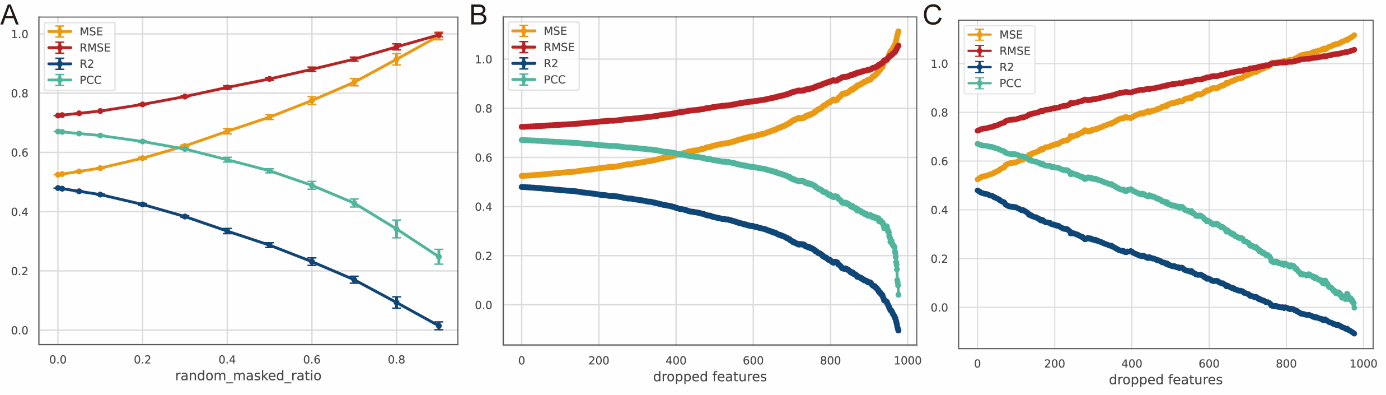
**

**Figure S4. Performance metrics of feature ablation. A** Random removal of increasing proportions of landmark genes. **B** Removal from lowest-ranked to highest-ranked features according to SHAP values. **C** Removal from highest-ranked to lowest-ranked features according to SHAP values.

**
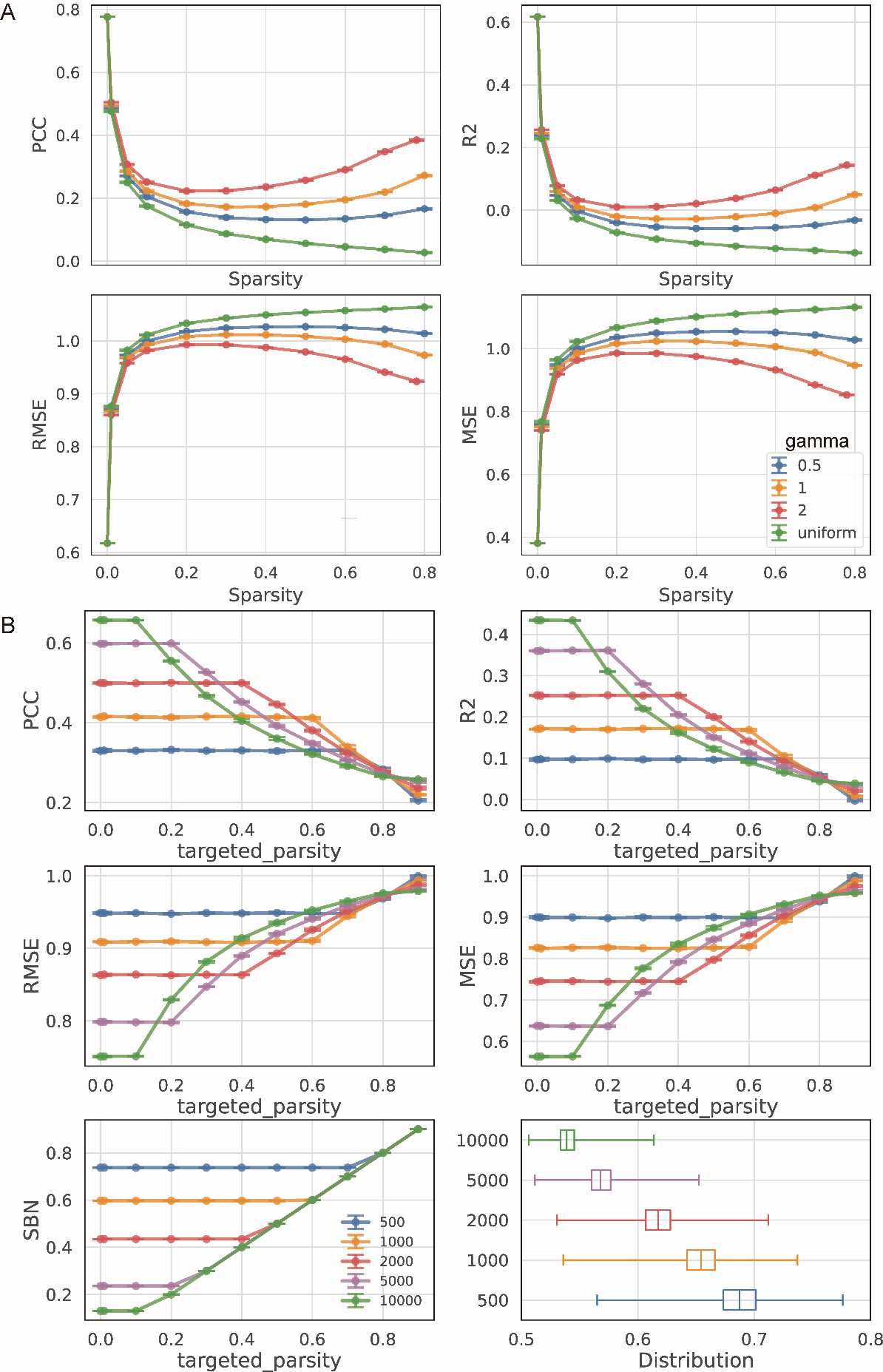
**

**Figure S5. Performance metrics under sparsity stress testing. A** Random and Poisson sampling to mimic expression-dependent dropout. **B** Multinomial UMI down-sampling followed by Poisson sampling, $\gamma$= 0.5. The boxplots show, for each sample, the distribution of expression-ranked genes that remain non-zero after multinomial sampling when at varying total UMI levels. SBN, Sparsity before standard normalization.

**
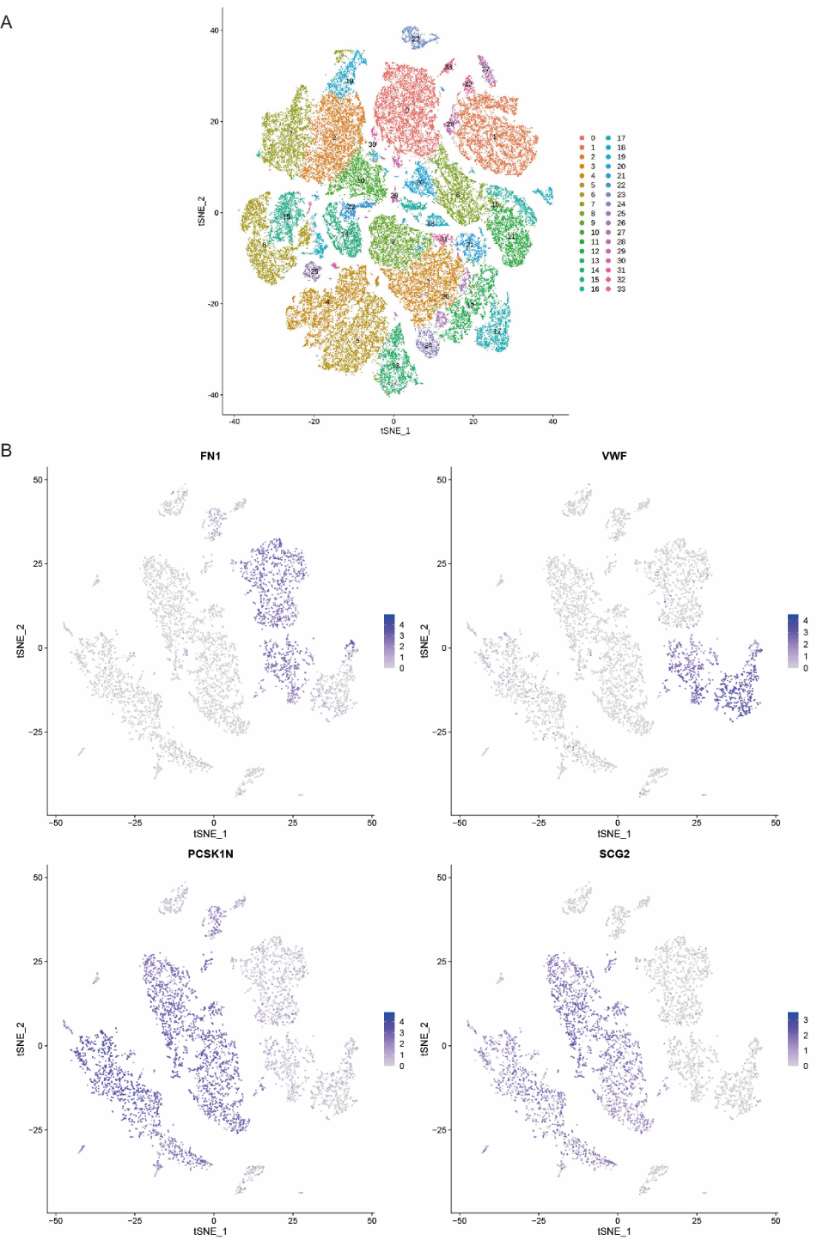
**

**Figure S6. T-SNE for scRNA-seq analysis. A** T-SNE distribution of different clusters of PDAC. **B** T-SNE distribution and cell-type marker expression among PA.

**
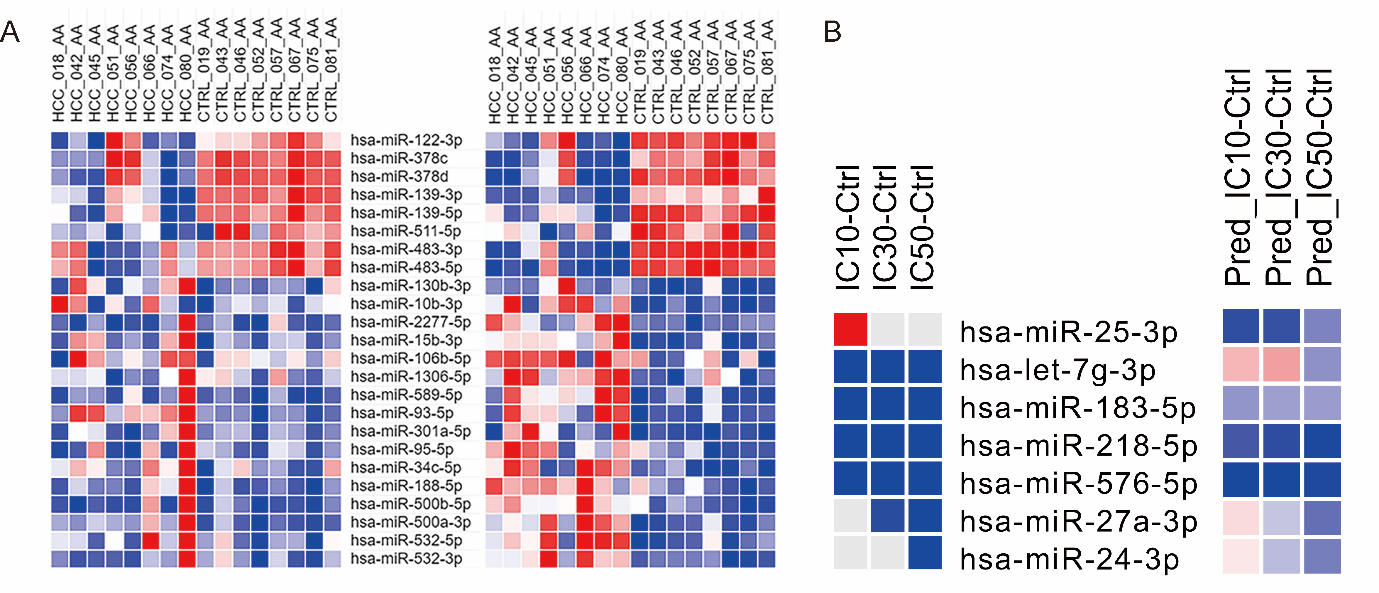
**

**Figure S7. Heatmap comparing predicted and real data of bulk RNA-seq for DEmiRs in A** Cancerous (HCC) and normal tissue (CTRL) from liver of African American population. **B** TCM-perturbated A549 cell line.

**
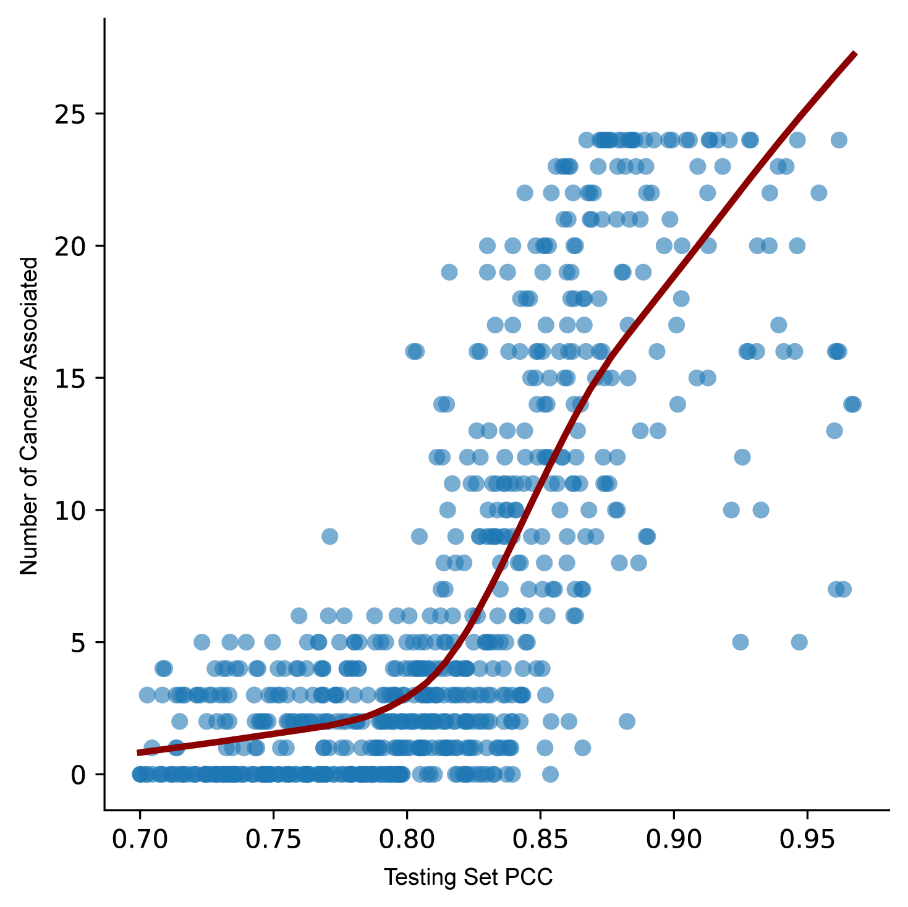
**

**Figure S8. Number of cancers related by each miRNA with PCC ranging 0.7-1.0.**


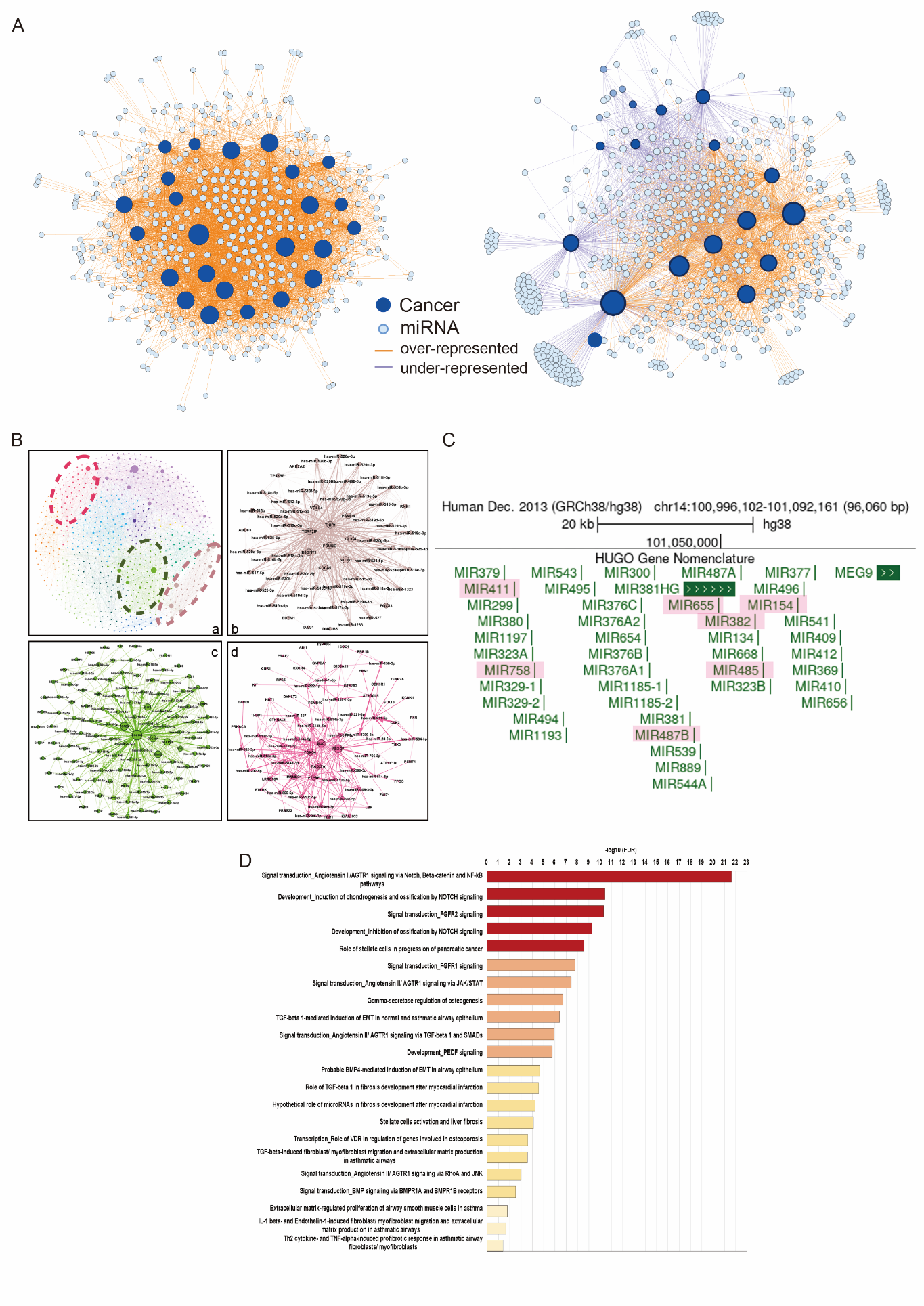


**Figure S9. miRNAs with PCC ≥ 0.8 have tighter association with cancer development, metastasis, and prognosis. A** Reported associations between miRNAs and cancers annotated by miEAA 2.0 retrieving from HMDD database are demonstrated in networks. Network (left) shows the associations between cancers and miRNAs with PCC ≥ 0.8. Network (right) shows the association between cancers and miRNAs with PCC < 0.8. Over-represented of miRNAs in cancers means the miRNAs over-expressed in cancer tissues, vice versa. **B** The contribution of landmark genes as features to expression of miRNAs are clustered into (a) 12 modules. (b-d)3 modules are visualized individually. **C** Host genes of miRNAs with whose expression positively contributed by COL1A1 locate densely at chromosome 14. **D** Enrichment analysis for Gene Ontology on target genes of miRNAs and contributing features.

**
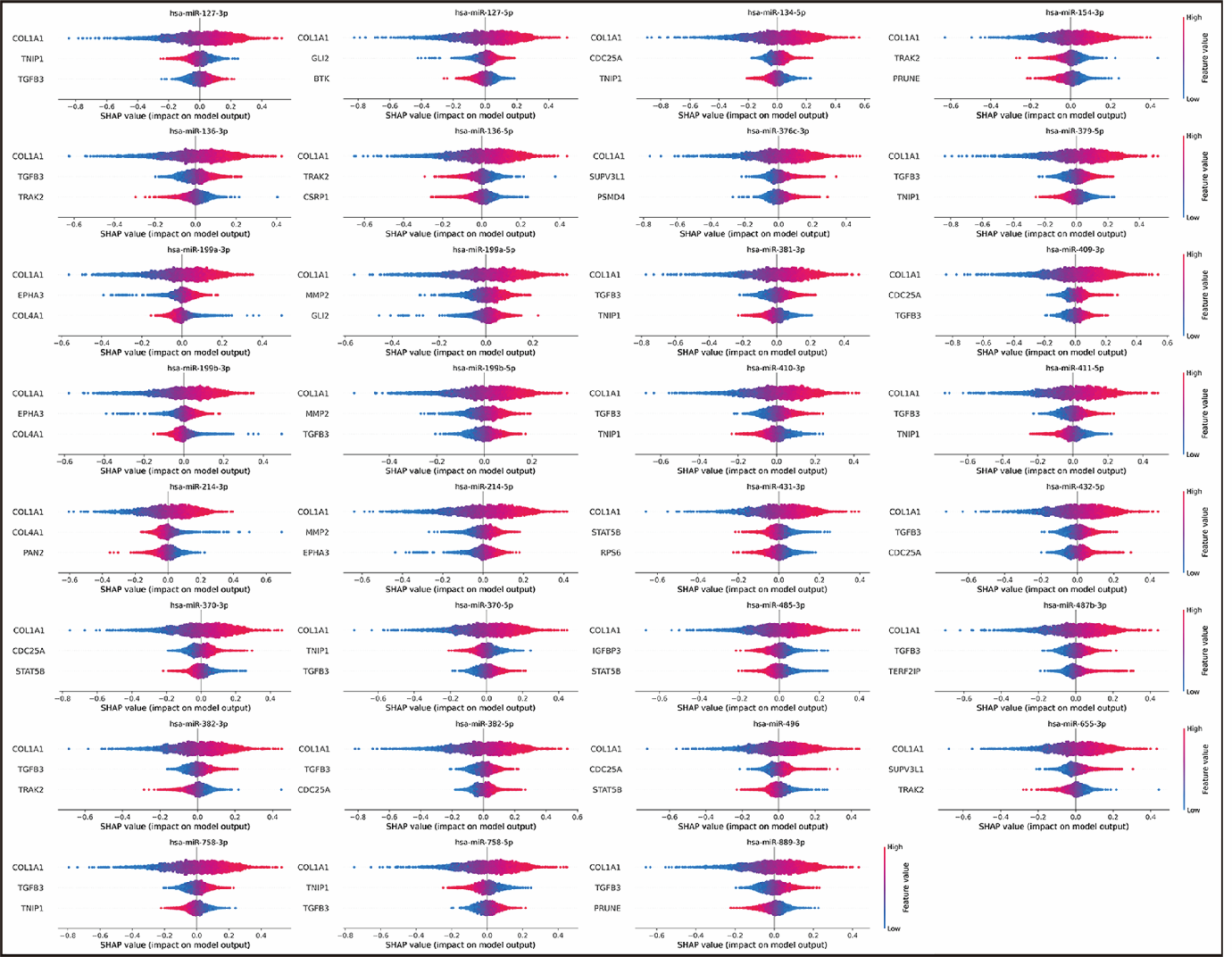
**

**Figure S10. SHAP analysis facilitated model interpretation and hub-miRNA discovery.**

**
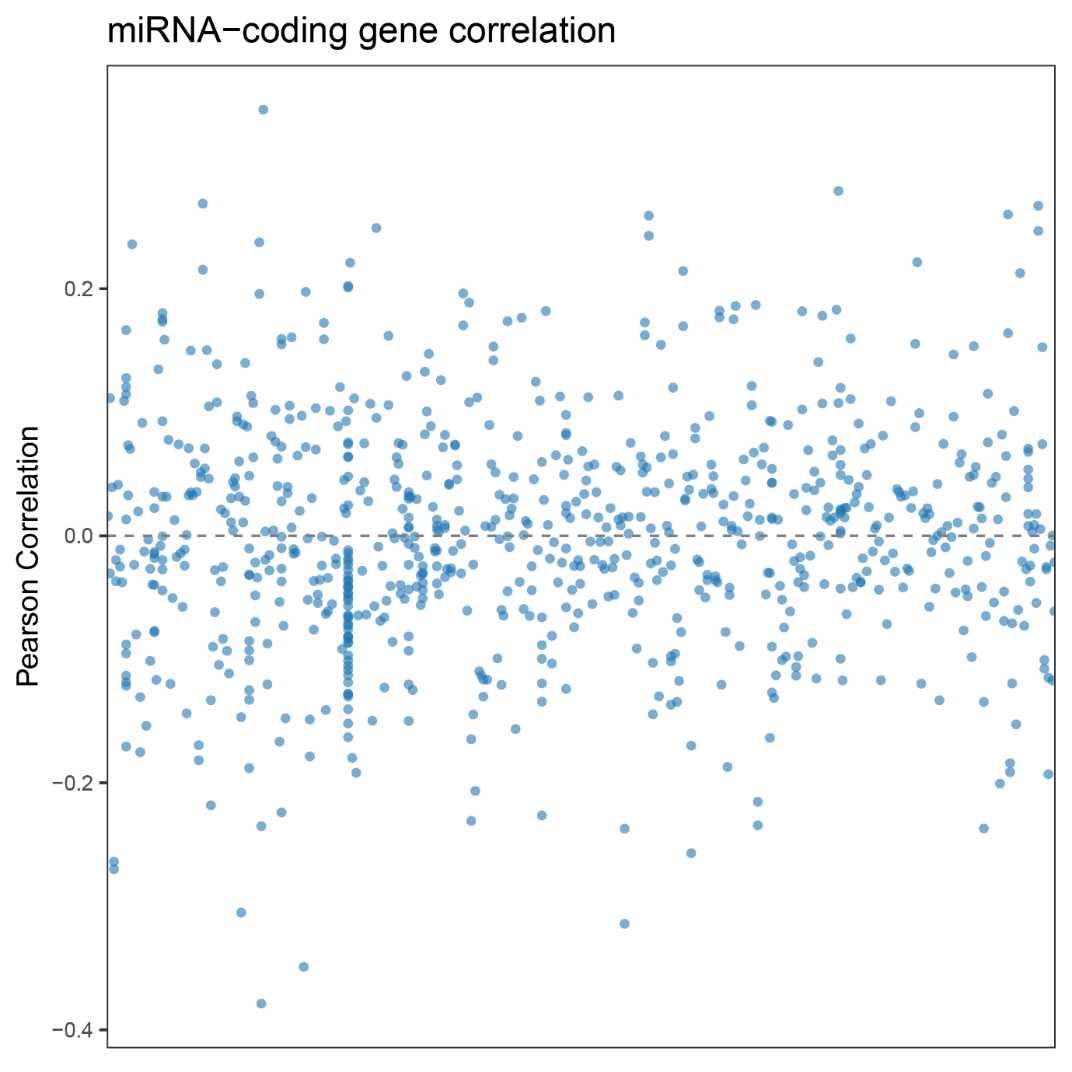
**

**Figure S11. Pearson Correlation Coefficient between mature miRNA expression and mRNA expression of miRNA coding genes in paired profiles from TCGA.** X-axis denotes miRNAs. Information of miRNA coding genes are retrieved from miRStart2.^[83]^

**
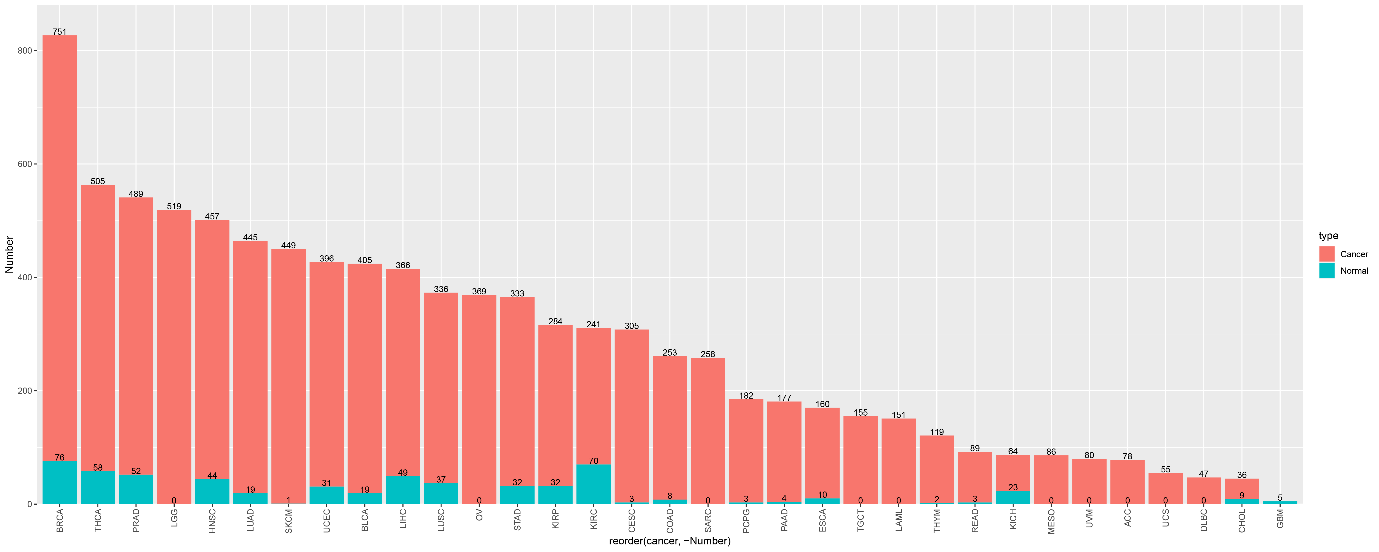
**

**Figure S12.** **Statistics of samples of cancers and corresponding normal tissues.**

**
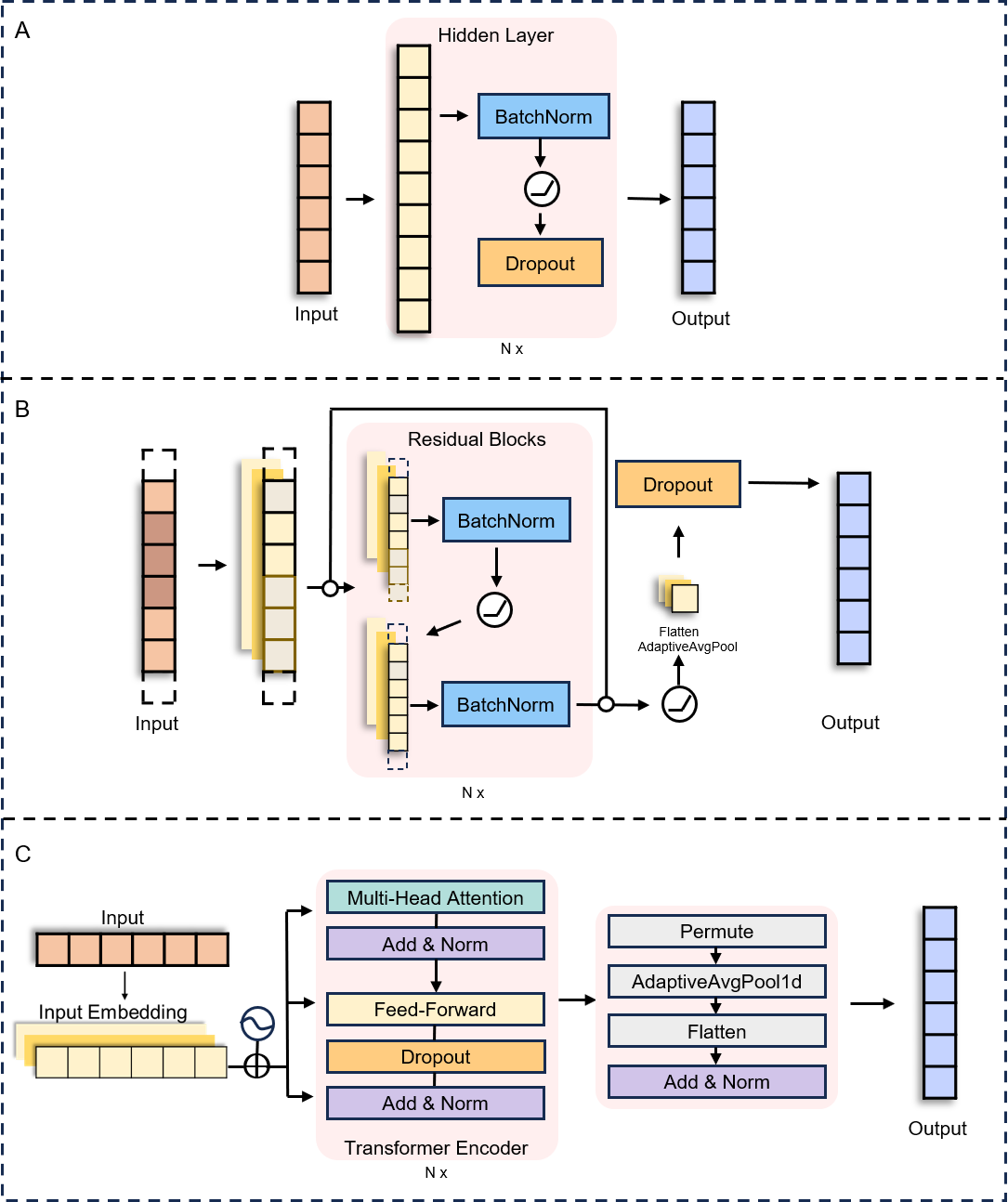
**

**Figure S13. Model architecture of models used for model constructure and comparison. A** Model architecture of (deep) neural network. **B** Model architecture of ResNet. **C** Model architecture of Transformer.

**
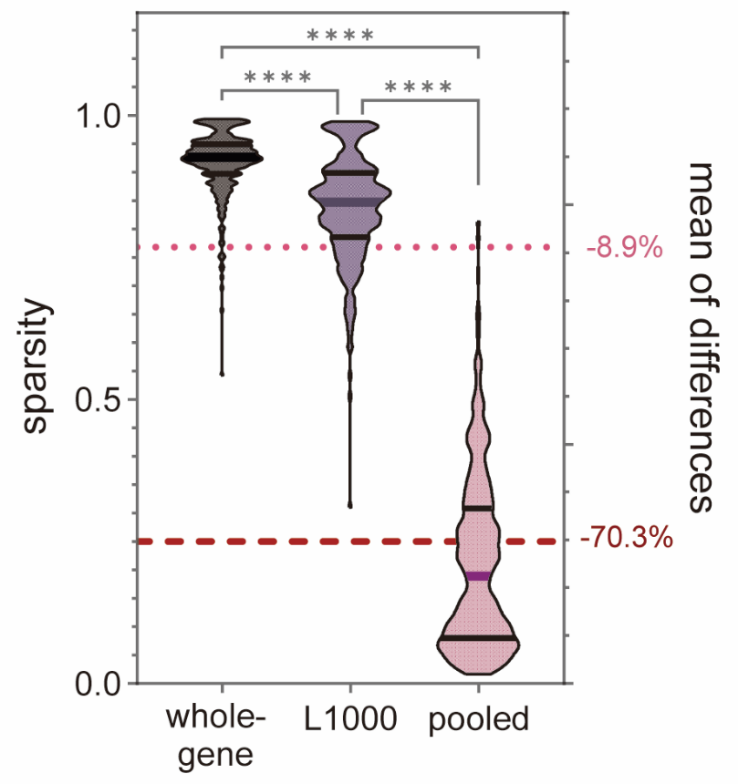
**

**Figure S14. Sparsity profiles of real single-cell expression data under feature extraction and pooling strategies.** Violin plots show the sparsity distributions of original whole-gene single-cell profiles (left), the subset of 977 landmark genes (middle), and SiCmiR-pooled cell-type averaged features (right). Dashed lines indicate the mean differences in sparsity relative to the whole-gene baseline. Statistical comparisons were performed using Wilcoxon rank-sum tests. ****P value < 0.0001.

**Table S1.xlsx.** Spearman correlation of miRNAs between miRSCAPE predicted and real TCGA-miRNA expression profile.

**Table S2.xlsx.** Data for application of SiCmiR in miRNA expression prediction and potential hub-miRNA discovery in PDAC.

**Table S3.xlsx.** Data for application of SiCmiR in miRNA expression prediction and potential hub-miRNA discovery in ACTH-secreting tumor data.

**Table S4.xlsx.** Fold change of bulk sequenced and predicted miRNAs in liver cancers and TCM treated A549 cell line.

**Table S5.xlsx.** Predicted PCC of miRNAs in GTEx dataset.

**Table S6.xlsx.** SHAP analysis, network analysis, and enrichment analysis data facilitate hub-miRNA discovery from the result of SiCmiR model.

**Table S7.xlsx.** EVmiR score, MTI score, and specificity of significant miRNA-target pairs from sender and receiver cells.

**Table S8.xlsx.** Search space of hyperparameters for model training.
